# An AI-powered smart Agribot for detecting locusts in farmlands using IoT and deep learning

**DOI:** 10.1038/s41598-025-23497-8

**Published:** 2025-11-13

**Authors:** Mana Saleh Al Reshan, Wahidur Rahman, Shisir Mia, Mehedi Hasan Talukder, Mohammad Motiur Rahman, Asadullah Shaikh, Tunc Asuroglu, Jawad Rasheed

**Affiliations:** 1 Department of Information System, College of Computer Science and Information Systems, Najran University, Najran, 61441 Saudi Arabia; 2 Emerging Trends and Technologies Lab (ETRL), College of Computer Science and Information Systems, Najran University, Najran, 61441 Saudi Arabia; 3 Department of Computer Science and Engineering, Mawlana Bhashani Science and Technology University, Tangail, 1902 Bangladesh; 4 Department of Computer Science and Engineering, Uttara University, Dhaka, Bangladesh; 5 Faculty of Medicine and Health Technology, Tampere University, Tampere, 33720 Finland; 6 VTT Technical Research Centre of Finland, Tampere, 33101 Finland; 7 Department of Computer Engineering, Istanbul Sabahattin Zaim University, Istanbul, 34303 Turkey; 8 Department of Software Engineering, Istanbul Nisantasi University, Istanbul, 34398 Turkey; 9 Applied Science Research Center, Applied Science Private University, Amman, Jordan; 10 Research Institute, Istanbul Medipol University, Istanbul, 34810 Turkey

**Keywords:** Agricultural robot (Agribot), Internet of things (IoT), Machine learning (ML), Deep learning (DL), Convolutional neural network (CNN), SVC feature selector, Computational biology and bioinformatics, Ecology, Health care

## Abstract

In many countries, locusts have significantly harmed agricultural production. To prevent their spread, the Agriculture Robot (Agribot) with cutting-edge technologies like the Internet of Things (IoT) and Machine Learning (ML) can be a possible solution. Thus, this study presents an astute way to develop an Agribot using IoT, ML, and DL-based architecture for detecting locusts in agricultural fields. The IoT framework ensures proper automation by utilizing various agriculture-related sensors, a centralized Android application, and an IoT cloud server. In contrast, the ML and DL methods include several pre-trained Convolutional Neural Network (CNN) models with conventional ML classifiers and a nature-inspired algorithm such as Artificial Bee Colony (ABC) and the SVC feature selector. To assess the proposed system’s efficacy, experimental data have been collected and interpreted accordingly. This research achieved the highest accuracy of 99.51% in locust detection using the VGG19 pre-trained CNN model with Logistic Regression (LR) and the SVC feature selector. In addition, the Agribot operated efficiently at a satisfactory speed in agricultural fields with live video streaming. The maximum speed of the Agribot was recorded at 0.3048 m/s. Furthermore, the study obtained a SUS score of 86% for the developed system. Although the system performs well in locust detection and automation in real field conditions, the research also identified some limitations during the study and implementation. However, the developed application demonstrates strong feasibility for real-time locust detection in agricultural fields.

**Supplementary Information:**

The online version contains supplementary material available at 10.1038/s41598-025-23497-8.

## Introduction

The Internet of Things (IoT) refers to a system linked with various types of networked devices and operational programming devices over the Internet. The IoT-based framework enables process automation, reduces waste, and improves services, lowering production and delivery costs and assuring customer interaction transparency^1^. The IoT has already had an enormous influence, particularly in the area of agriculture^2^. Through the extensive use of a wide variety of agricultural sensors and microcontrollers, the IoT makes a significant contribution to the management of waste, as well as to smart irrigation, crop cultivation, weed monitoring, pest prevention, and even in the development of Agricultural Robot (Agribot)s^2^. Agribots are particularly efficient in farmers’ occupations, boost processing quality, and minimize the industry’s reliance on manual labor^3^. There are numerous types of Agribots, including (1) Demeter, (2) Weed control robots, (3) Forester robots. (4) Horticulture robot, (5) A robot that picks fruit. These automated systems significantly minimize human labor in harvesting, smart irrigation, seed distribution, weed control, and even crop mashing. On the other hand, Machine learning (ML) and Deep Learning (DL) have been used in novel ways to help farmers and agricultural employees improve early detection of pests and diseases, crop yields, and crop quality^4^. In addition, ML and DL techniques show immense promise for usage in agriculture, where they could supply critical information such as soil quality, optimal planting and spraying timings, and pest-infested places. These strategies have been used all across the world to help farmers keep an eye on their crops^5^. A large range of ML and DL-based solutions have been created to provide farmers with data-driven decision-making assistance tools. This phenomenon will help farmers safeguard and improve crop quality and yield while reducing the pesticides they need. So, any real-world solution paired with IoT and ML, particularly in agriculture, will provide incalculable results.

Again, crop production consistently faces multiple challenges from infectious diseases, insects, and pests. Plant diseases and pest infestations account for 20–40% of global agricultural production losses, with estimated costs to the global economy ranging from $220 billion to $70 billion yearly^6^. There are many different kinds of destructive insects in the world, but locusts are the most prevalent kind that damages crops and even green leaves. The locusts are various species of short-horned grasshoppers of the *Acrididae* family. Different types of locusts are found worldwide, including Desert locusts, Migratory Locusts, the Australian Plague Locust, the Red Locust, and the Rocky Mountain Locust. No taxonomic distinction exists between locust and grasshopper species; the classification is based on whether a species creates swarms under intermittently favorable circumstances^7^. In addition, when there are appropriate conditions of drought followed by rapid vegetation growth, the production of serotonin in their brains triggers a significant sequence of transformations: they begin to reproduce in large numbers, becoming social and migratory when their populations reach a high density. They organize into groups of wingless nymphs, which subsequently transform into swarms of winged adults. Both the bands and the swarms of insects fly around and rapidly remove vegetation from fields, resulting in crop damage. The adult insects possess exceptional flight capabilities, enabling them to cover vast distances. They consume substantial amounts of green vegetation in the areas where the Swarm settles^8^. These insects are generally solitary, but they grow more numerous in certain situations, change their behavior, and become more gregarious^8^. Locusts are found all across the world, although they are particularly devastating in Africa’s subsistence agriculture zones. In Somalia, a national emergency has been declared due to the swarming. Ethiopia and Kenya are trying to keep the pandemic alive, and swarms have moved over the Arabian Peninsula and onto both sides of the Persian Gulf^8^. Furthermore, desert locusts can readily move with the wind and traverse more than 90 miles each day, often traversing the Red Sea, which is 186 miles wide, with the capacity to stay in the air for extended periods^8^. Therefore, early detection of locusts in agricultural fields can reduce crop destruction and save the greatest quantity of food and harvesting.

Thus, the study aims to examine and determine the agricultural scope of IoT and ML-based Agribot. It also explores the intriguing issues, difficulties, and practical implications for decision support, information management, and the deployment of farmer and regular user assistance systems. This study made the following contributions:

- To develop an Agribot using the principles of IoT and ML approaches identify locusts in agricultural fields.
- To implement and use the number of cloud-based IoT sensors used to monitor the status of farm.
- To efficiently identify the locust using the core machine learning principles and a cloud-based nature-inspired algorithm such as Artificial Bee Colony (ABC).
- To combine all aspects in order to achieve total automation in the agriculture industry.

The manuscript is classified into 5 five sections. Section two describes the background study of the previous works related to this research. Section three reflects the proposed methodology and the working principles of the evert modules of the research. Section fours provides the results associated with this research. Finally, section five illustrates the conclusion with the existing drawbacks and future scopes.

## Related studies

This section provides a contextual study of the existing solutions. Many great contributors have placed some significant contributions in the field of Locust detection, more specifically, locust detection and classification with Machine Learning (ML) and Deep Learning (DL) along with Internet of Things (IoT) embedded systems.

### Literature search method

**Fig. 1:**
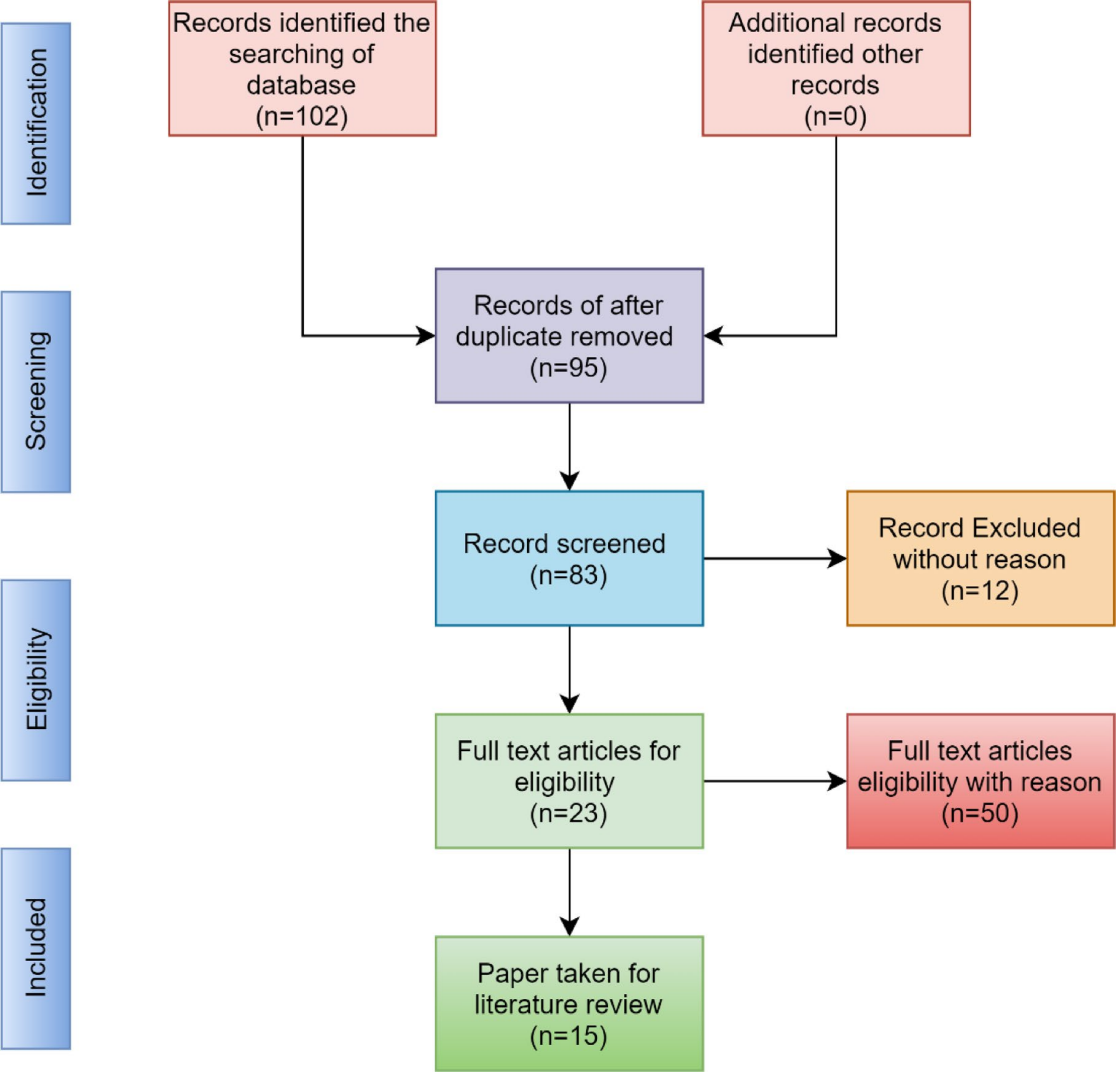
Search criteria of literature reviewing.

This research reviewed the literature on four interconnected parameters: identification, screening, eligibility, and inclusion. Figure 1 shows the corresponding literature review methodology of this manuscript.

In this Figure, the research has performed identification on the records of 102 articles. After that, a screening procedure is performed to find duplicate papers. Ninety-five papers are extracted from the screening procedure. Then, we achieved an eligibility operation on the exclusion criteria. Finally, we have taken 15 articles for the literature review.

### Literature reviews

An insect damage detector trained on two distantly related plant species (*Quercus bicolor and Onoclea sensibilis*) was used to automate herbivorous data extraction from herbarium specimens^9^. While detecting and classifying two types of damage simultaneously, accuracy was mixed at 45%. These models are being improved so researchers may extract and apply similar data to their biological assumptions. The paper^10^ explored the technology and scientific states-of-the art of using sensors to detect and monitor insect pests automatically. The report focused on the methods of recognizing infrarouge sensors, sound sensors, and image classifications based on pests, presenting the various systems available, implementation examples, and emerging technologies, like machine learning or the IoT. Besides, the paper^11^ evaluates supervised machine learning such as Linear Discriminant Analysis (LDA), Decision Trees (DT), Support Vector Machine (SVM), K-Nearest Neighbors (KNN), and Naïve Bayes (NB) for recognizing mosquitoes using optical signals. The algorithms were trained to identify mosquito species, sex, and gravidity in New Jersey, USA, utilizing predictor variables from wingbeat frequency and optical cross-section. They found that the Support Vector Machine is the best machine learning method for mosquito identification, performing 0.65 to 7.3% better on all tests. The Support Vector Machine outperformed the second-best algorithm for complicated tasks by almost 2%. The best algorithm for real-world study with several mosquito species at one site was it. Linear Discriminant Analysis, which was 0.65% better than all Support Vector Machine jobs and most efficient for mosquito gravity, came a close second. Decision Trees was practically flawless at determining the sex of a single mosquito species with 99.9% precision, 1.3% better than the second-best algorithm.

Besides, the authors of the paper^12^ addressed feed-forward neural network (FNN) training challenges by applying a newly created meta-heuristic Locust Swarm Optimization (LSO). Their method was used in a series of experiments to examine the proposed approach’s capabilities and performance. According to the paper^13^, the ResNet-Locust-BN model leveraged with CNN to identify locust species and instars. A BatchNorm function beyond each convolution layer improves the network’s stability, convergence speed, and classification accuracy in this ResNet-based model. The model was trained using the collected locust images from the field. After testing the activation function, starting learning rate, and batch size, the author had utilized ReLU. Experimental results reveal that the ResNet-Locust-BN model can distinguish AM locusts from rice and cotton locusts (93.60 and 97.80% accuracy, respectively). The model identified AM locust growth state details with 90.16% accuracy (third-instar (77.20%), fifth-instar (88.40%), and adult (93.80%). This is higher than AlexNet (73.68%), GoogLeNet (69.12%), ResNet18 (67.60%), ResNet50 (80.84%), and VGGNet (81.70%).

On the other hand, the authors of the paper^14^ developed a control mechanism to position the seed at a given distance from two seeds to the seedlings when they were planted. This multipurpose device provides an advanced method for weaving, plowing, and watering crops with minimum human power and labor to make it an effective vehicle. The computer can cultivate the field by considering individual rows and particular columns at a fixed distance depending on the crop. Besides, the paper aims to use emerging technology, i.e., IoT and Bluetooth, resulting in Smart Farming. The entire measurement, collection, and monitoring process is built with microcontroller-interfaced motors & sensors. Further, the article^14^ proposed an intelligent Agribot water frame system intended to reduce water and increase supplies of crops using the Internet of Things (IoT) for agricultural industries. The proposed IoT-Agribot would raise the water framework, increase cost-effective water use, and cut the workforce to precision farming. The proposed system^15^ aimed to design autonomous agriculture Robotic multifunctional vehicles for crop plowing, seed sowing, and irrigation. This robotic vehicle was a farming machine with substantial power and a high degree of soil clearance. The author of the paper presented IOT based grass cutter required for cutting grass in the gardens. Various sensors helped to find unwanted obstacles in the field. The proposed model was implemented with Arduino, LCD display, motor driver, and power supply circuit. The LCD was used for better-receiving commands from the user. A Solar grass cutter was a machine that cut grasses of equal length. The paper’s authors proposed an important analysis of different methods for selecting crops, sowing crops, weed detection, and device monitoring to generate a profitable production. The article^16^ aimed to design, grow and produce the Agribot, a multipurpose bot capable of carrying out all farming operations, including plowing field soil, plugging field seeds, planning the field by use of the plowing leveler, watering crops, fertilizing and camera-controlled Agribot. The project^16^ was intended to create and produce an intelligent seed plant robot for agricultural purposes. This intelligent seed robot included a robot arm to seed the agro plants from the container seeds. To categorize desert locust presence, the author^17^ introduced new data from the SMAP satellite and used a variety of machine learning approaches in our species distribution model. The authors achieved relatively satisfactory model results for identifying the possibility of availability and potential breeding areas based on environmental parameters (KAPPA & TSS = 0.901 and ROC = 0.986). The most critical variables under varied time scenarios were surface temperature, NDVI, and root zone soil moisture. In addition to strengthening MODIS’ NDVI product as a trustworthy environmental predictor, this study demonstrated the SMAP satellite’s capacity to retrieve key temperatures as time progressed.

Based on the discussion, the authors of the papers discussed papers had only focused on developing insect detection mechanisms and processes using machine learning and deep learning. One of the articles developed an agricultural robot with IoT and robot. Still, no strategy had been adopted to detect and monitor Locust in real-time using modern technology such as IoT cloud servers, deep learning, and Android application. But, this work demonstrates an intelligent approach to developing an Agribot for identifying locusts in the agricultural field using IoT, ML, and DL-based architectures. The IoT component offers the right automation by utilizing numerous agricultural-related sensors, a centered Android application, and an IoT cloud server. The ML and DL technique, on the other hand, incorporates some pre-trained Convolutional Neural Network (CNN) models with traditional ML classifiers and a nature-inspired algorithm like Artificial Bee Colony (ABC) and SVC feature selector. Thus, the proposed solution provides complete automation with IoT and an effective way to detect locusts in the agricultural field.

## Proposed methodology

**Fig. 2:**
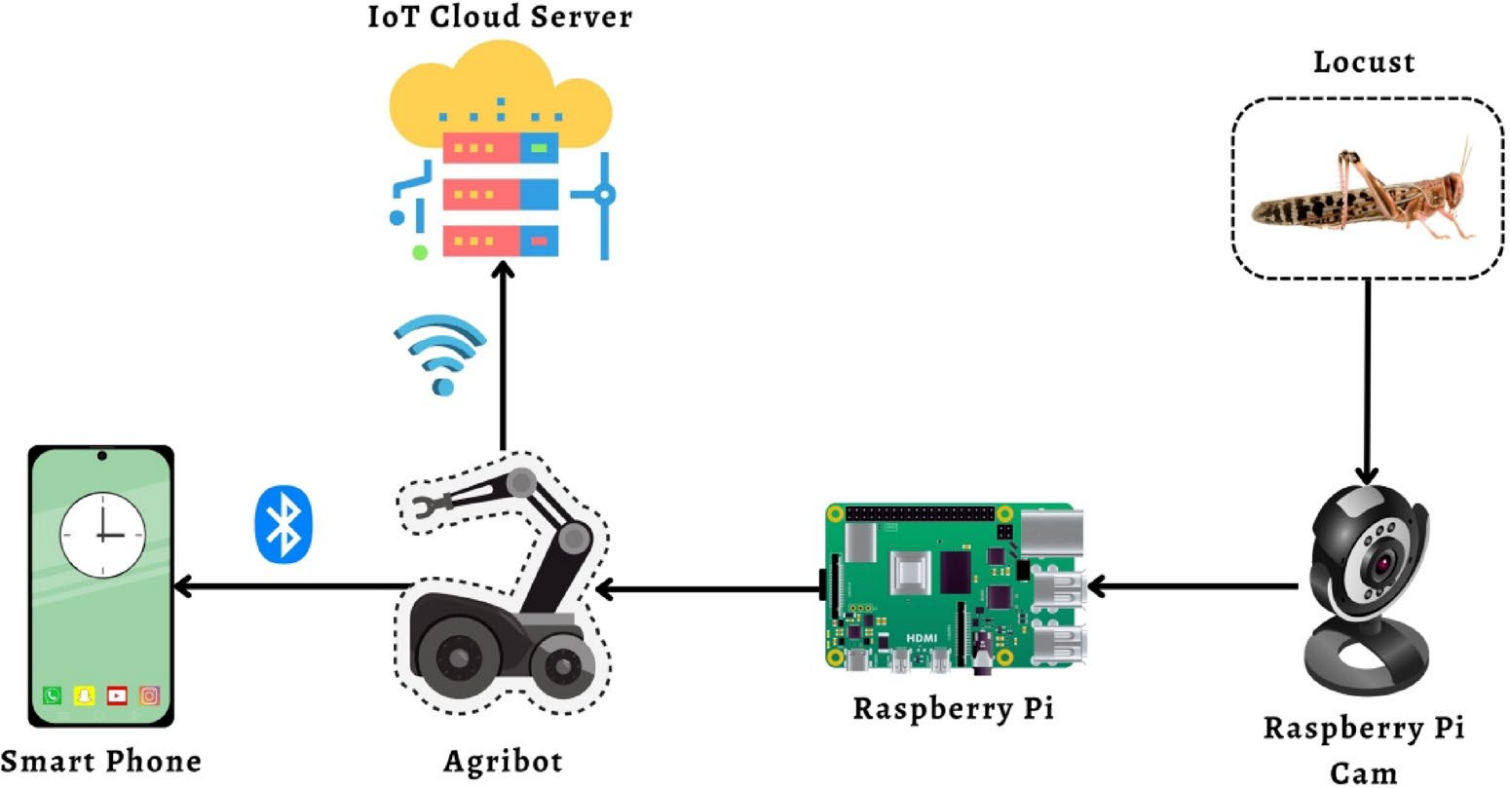
Entire block diagram of the proposed system.

This section is classified into three interconnected sub-sections. The first part describes the working principles of Machine Learning (ML) and Deep Learning (DL) and associated observable annotations. The second part provides the operational principles of the IoT-based solution, along with its orientation and embodiment. Finally, the third presents the working procedure of IoT-based cloud servers^18^. Figure 2 shows the overall graphical abstract of the proposed solution.

The Figure is partitioned into four interconnected sections. In the first portion, the locust detection criteria are depicted. Raspberry Pi and a camera module are mounted to detect the insect^19^. The Raspberry Pi camera module is responsible for detecting locusts in the agricultural field with the aid of Raspberry Pi. The Raspberry Pi processes the detected information to the Agribot. Secondly, An Agricultural Robot is set for the right movement in the field. Thirdly, a smartphone application is implemented to monitor and operate the Agribot and overall system, where the app is connected to the system through Bluetooth. Finally, a cloud server is also implemented to check the data monitoring when connected with a cloud server through Wi-Fi.

**Fig. 3:**
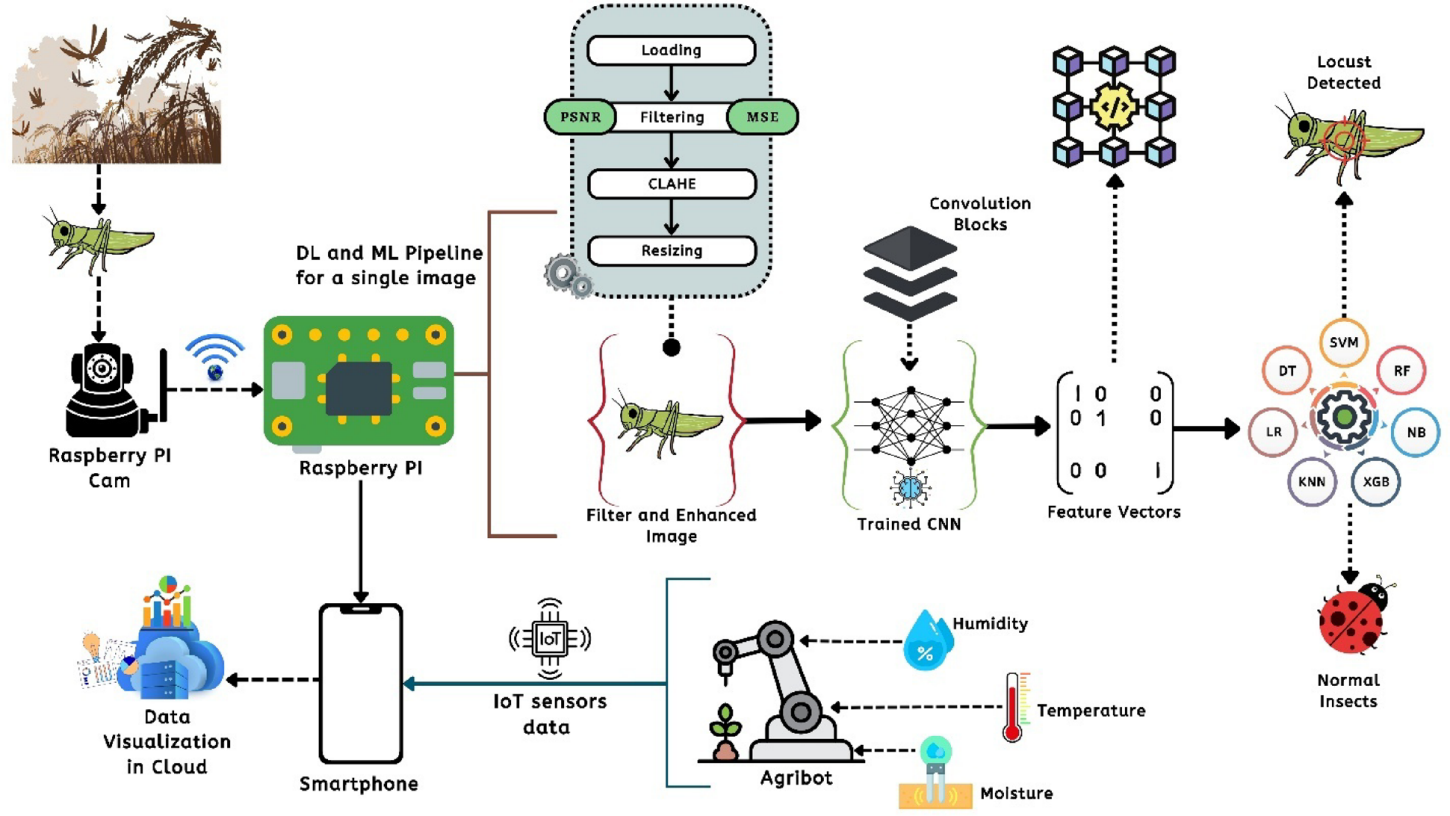
System flowchart with DL + ML pipeline for a single and how the system works in the agricultural field while detecting locusts along with its IoT based sensors.

Figure 3 shows the breakdown of the orientation of the proposed model including the integration of the DL and ML methods after capturing an image along with the IoT orientation. In this figure, after system detecting the single image of the locusts, it sends to the raspberry Pi. The Pi then applied some image processing techniques. The trained model then applied to extract the features and on the extracted features ML classifiers have been applied to find the decision whether the locust has been detected or not. Also, the figure also suggests the orientation of the IoT sensors data and visualization of the IoT data in the cloud.

### The working principles of ML and DL

**Fig. 4:**
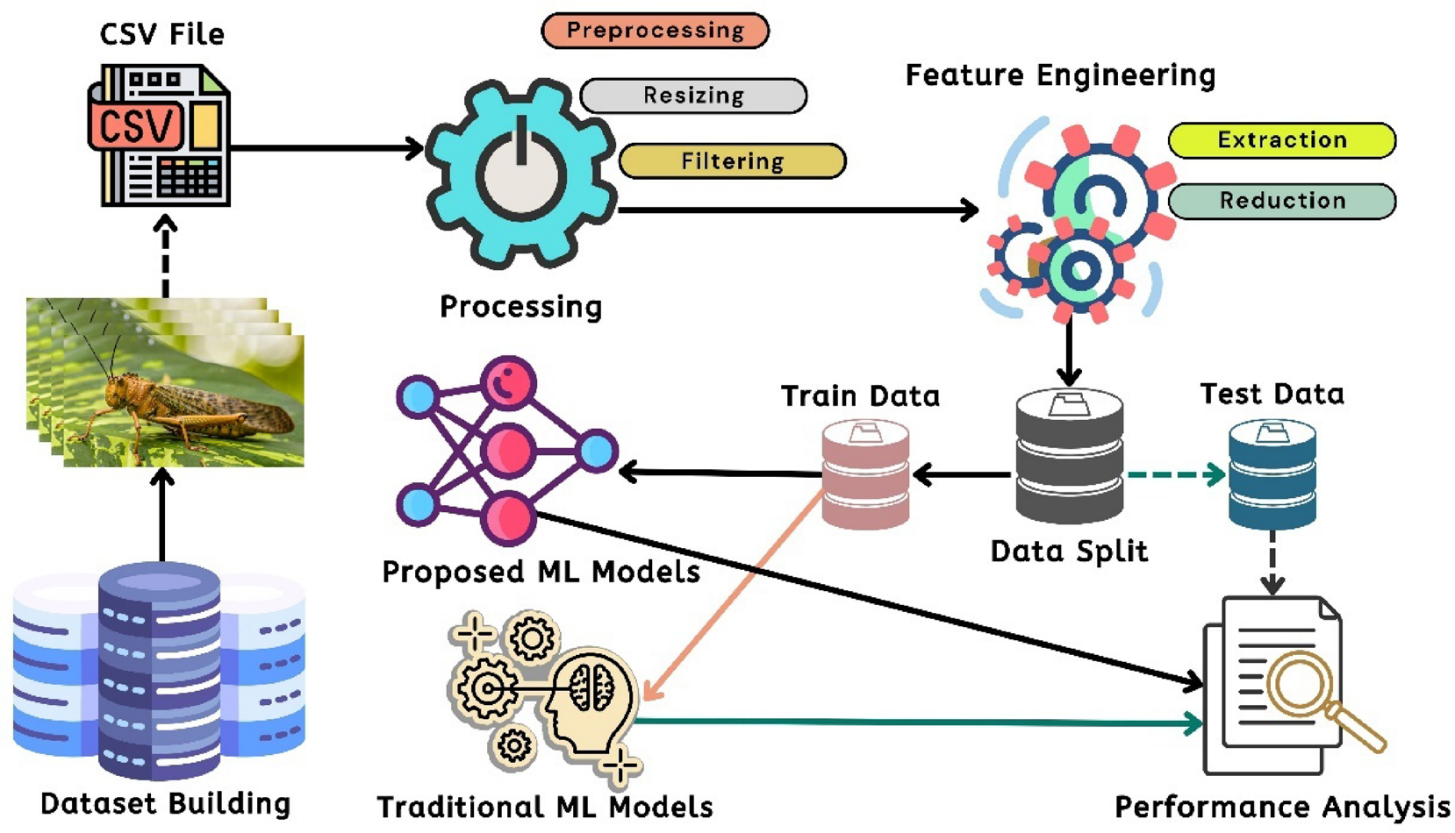
DL and ML-based pipeline for Locust detection with how the research classified the dataset in training and test data.

This section reflects the proposed basic interpretations of Machine Learning (ML) and Deep Learning (DL) based pipelines. Figure 4 shows the corresponding pipeline for locust detection. In this diagram, the dataset is initially gathered from secondary sources. Following that, a set of images pre-processing such as filtering based on the value of the Mean Square Error (MSE) and Peak Signal to Noise Ratio (PSNR) was used to enhance the dataset. The preprocessed and filtered images are then fed into pre-trained Convolutional Neural Network (CNN) models, which extract the key attributes from the signal image. Then, the research used feature-optimization techniques to extract the best characteristics from the obtained imagery features^20^. Subsequently, the dataset is divided into two identical portions: training data and test data, with the training data utilized by machine learning models employing established conventional methods to obtain experimental results. Test data has been utilized to evaluate performance analysis metrics. Subsequent to obtaining the experimental results, a set of assessments are conducted.

The images are collected from secondary data sources like Mendeley data, Kaggle, DataIo and IEEE data. This research mainly contains images of three identical species. Table 1 shows the corresponding samples of the collected Locust Species. In this table, it clearly shows that we have collected mainly three species of the locust images such as Desert Locust, Australian plague locust, Migratory locust. After collecting the locust images, we have collected the images of the agriculture field. These agriculture field’s images include the paddy field, tomato field, jute field and potato field. Thus, the images for this research are classified into two identical classes: with-locust and without-locust. More than 1000 + images have been used to extract the features where 505 belongs with locust and the rest of the images fit for the without locust classes. Table 2 shows the corresponding experimental images for extracting the features.

**Table 1:** Sample images of three species of Locust.

Species name	Desert Locust	Australian plague locust	Migratory locust
Samples			
		

**Table 2:** Sample images of locust to build the ML models.

Species name	With locust	Without locust
Samples		
	

#### Method for extracting features from CNN models

**Fig. 5:**
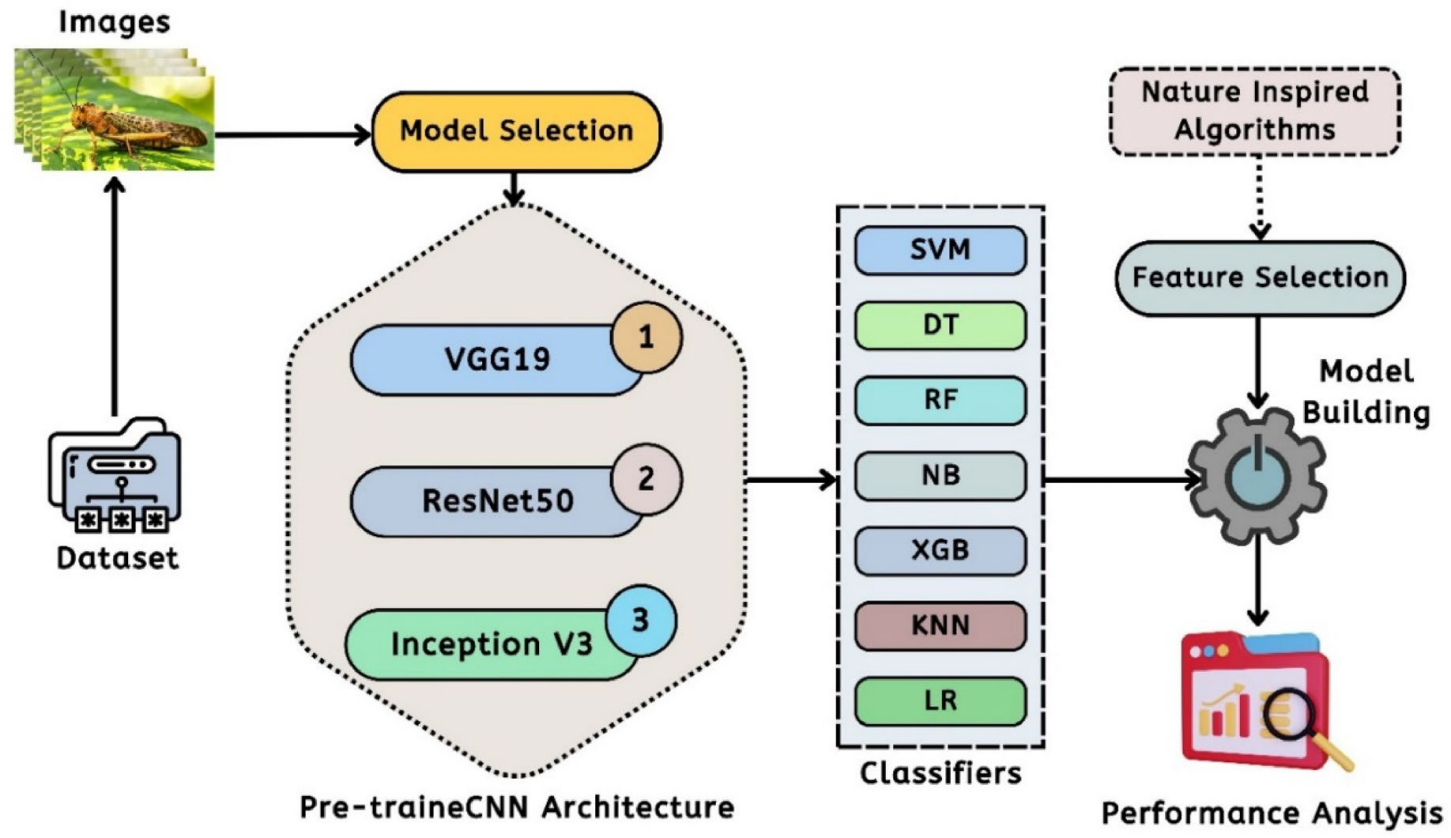
The proposed DL & ML based pipeline for feature extraction from a individual image of the locust with pretrained models as DL models and ML classifiers^21^.

This study has utilized three well-established pre-trained CNN models to extract features from an indivosual image of a locust. Figure 5 illustrates the framework of the respective pipelines for feature extraction techniques in this study. In this diagram, the system initially extracts files from the dataset to provide the pre-trained models with the images^20^. Three standard pre-trained CNN architectures were employed in succession to derive feature vectors from images: VGG19, ResNet50, and InceptionV3. Seven standard classifiers were employed to classify the images after the feature extraction process. To utilize the most effective features, algorithms inspired by nature have been implemented, and the approach for feature selection has been carried out. Subsequently, the system assesses performance according to their primary categories. The entire pipeline employs Algorithm 01 to derive feature vectors from a designated image.

**Algorithm 01:**
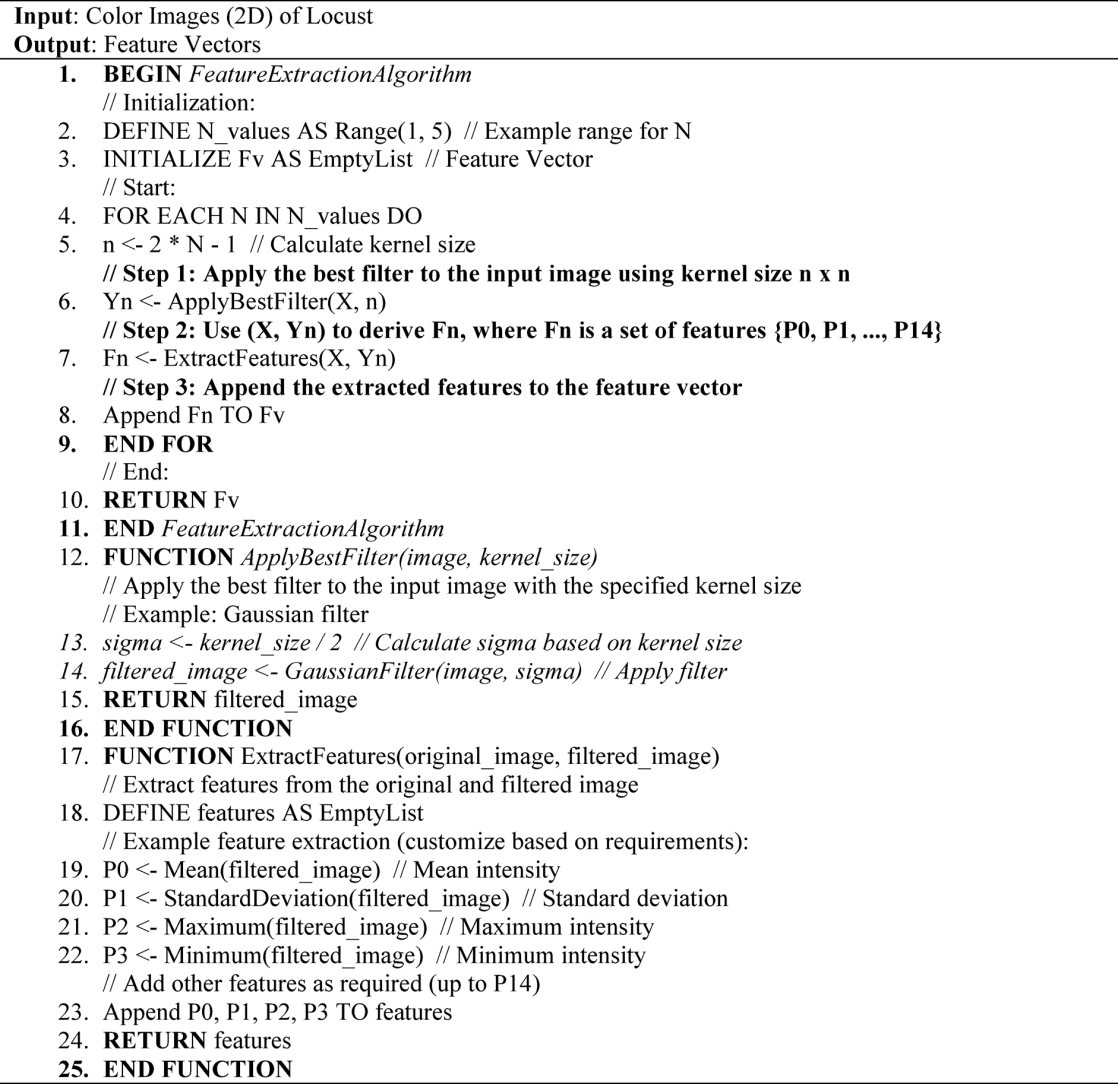
Procedure for extracting feature vectors using the suggested pipeline.

**Fig. 6:**
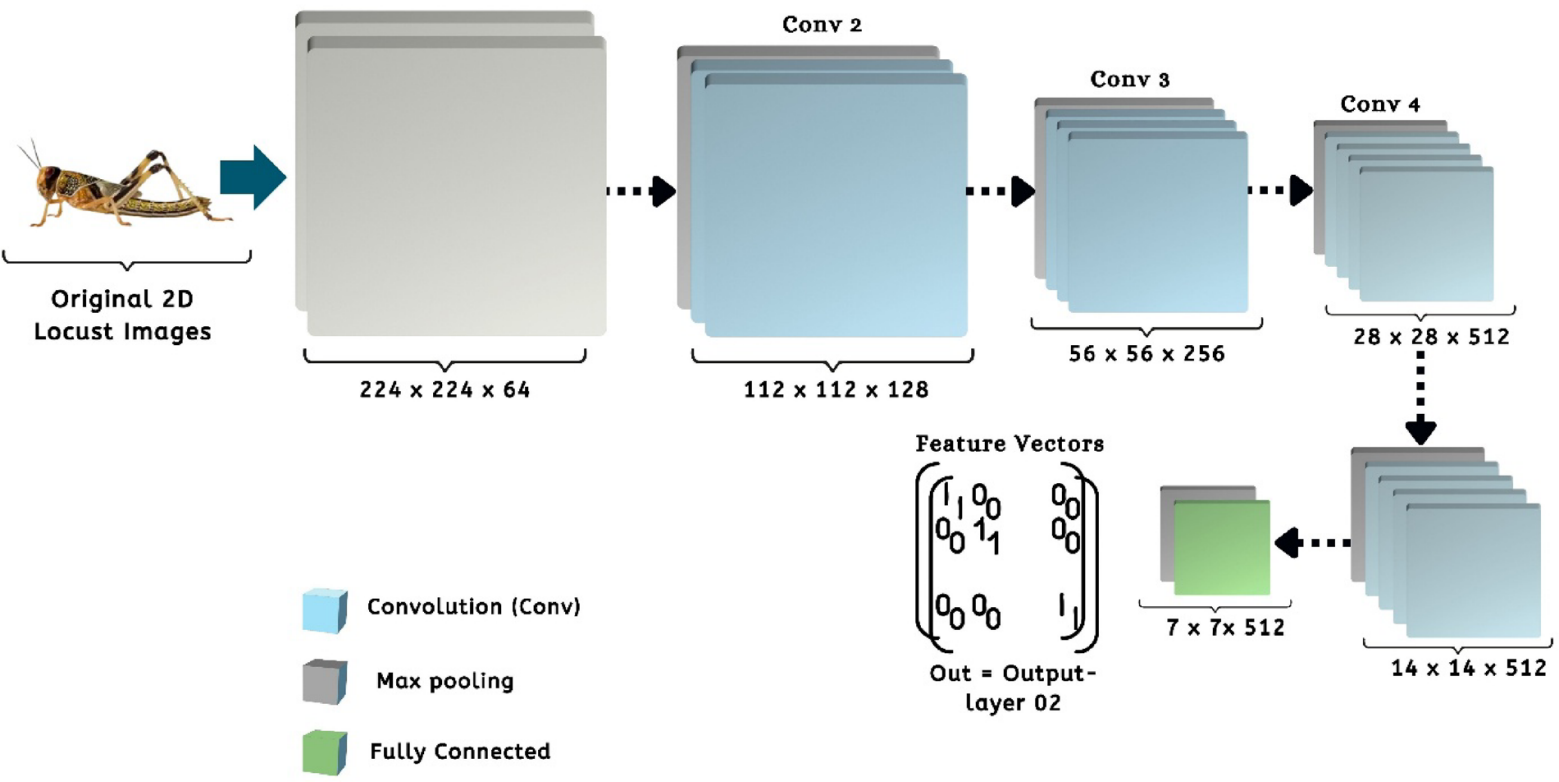
The corresponding architecture of the VGG19 pre-trained CNN model to extract the feature vectors by using layer-02^21^.

**Fig. 7:**
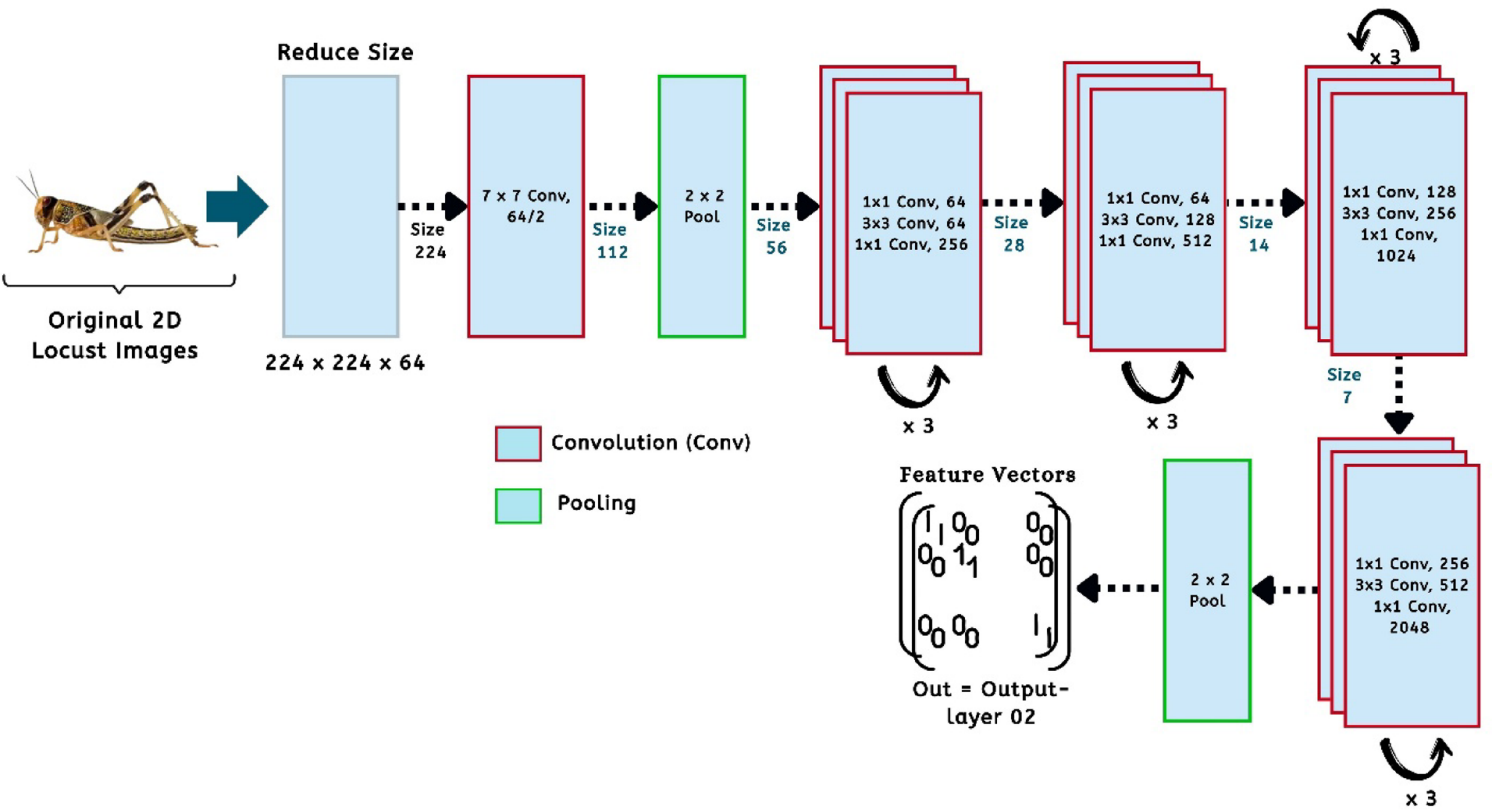
The architecture of the proposed ResNet50 pre-trained CNN model for our feature extraction using layer-02.

Additionally, this study outlines the framework of the pre-trained CNN model that has yielded promising results in locust detection. Figure 6 illustrates the architecture of the VGG19 model within our proposed machine learning pipeline. Using a limited dataset, VGG19 is adjusted by modifying certain layers to mitigate the risks of overfitting^21^. This architecture features a pre-trained model that includes a series of Convolutional Layers (CL) along with one or more Fully Connected (FC) layers. The model consists of two interrelated sections. The initial segment illustrates the process of feature extraction, progressing from the input layer to the concluding max-pooling layer. The second section illustrates the residual network of the model, which plays a crucial role in the categorization process. The proposed approach focuses on utilizing the VGG19 model mainly for feature extraction, thereby omitting the classification component in this investigation. The model employing VGG19 processes 224 × 224 × 3 locust color images and extracts 4096 significant features from the final layer of the feature extraction component for each image^21,22^. Again, the pre-trained ResNet50 CNN architecture has been utilized in this study to extract features from individual image data. ResNet50 consists of 50 layers and approximately 2 million parameters. The initial segment comprises 64 kernels, incorporating a max-pooling layer, a convolution layer, and a fully connected layer. The augmentation layer allows for the occurrence of degradation issues and addresses the problem of disappearance. This study employs ResNet50 solely for the purpose of feature extraction, without incorporating a classifier. The ResNet50 model in this framework takes an input image size of 224 × 224 × 3 and produces an output of 2048 features from the final layer of the feature extraction section for each image. Figure 7 illustrates the architecture of the associated pre-trained CNN model^21^. The input image for the model is 224 × 224 pixels, as illustrated in this figure. The convolution was then occupied by 50 layers and 20 million parameters, utilizing 64 kernels and fully connected layers. The models generate the features of 2048 from each locust’s images^23^.

#### Feature selection with SVC

Linear Support Vector Classifier (SVC) deals with the specific problem. The SVC’s overall purpose is to fit the data and deliver the “best feature” hyperplane to categorize the data from a given dataset^24^. The SVC feature selection in the proposed model operates as shown in Algorithm 02.

**Algorithm 02:**
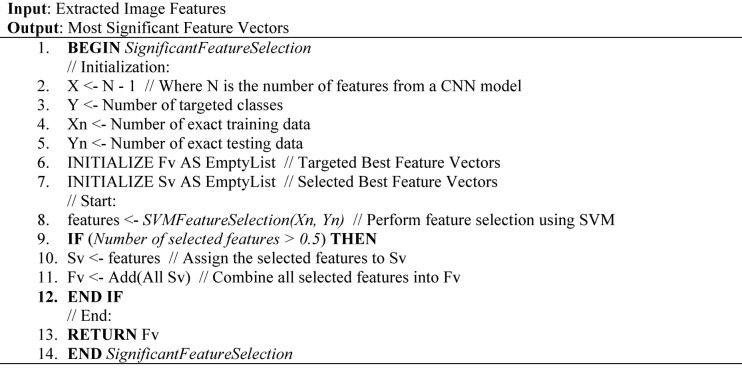
Working mechanism proposed SVC in feature selection.

#### Classifiers

This paper uses ML approaches to extract characteristics from instances of Locusts. The study employs well-known classifiers present in contemporary ML practice^21^. The final model is built using seven different classifiers, including Support Vector Machine (SVM), Random Forest (RF), Decision Tree (DT), Naive Bayes (NB), Extreme Gradient Boosting (XGB), K-Nearest Neighbor (KNN), and Logistic Regression (LR)^25^.

#### The application of nature-inspired algorithms in feature selection

**Fig. 8:**
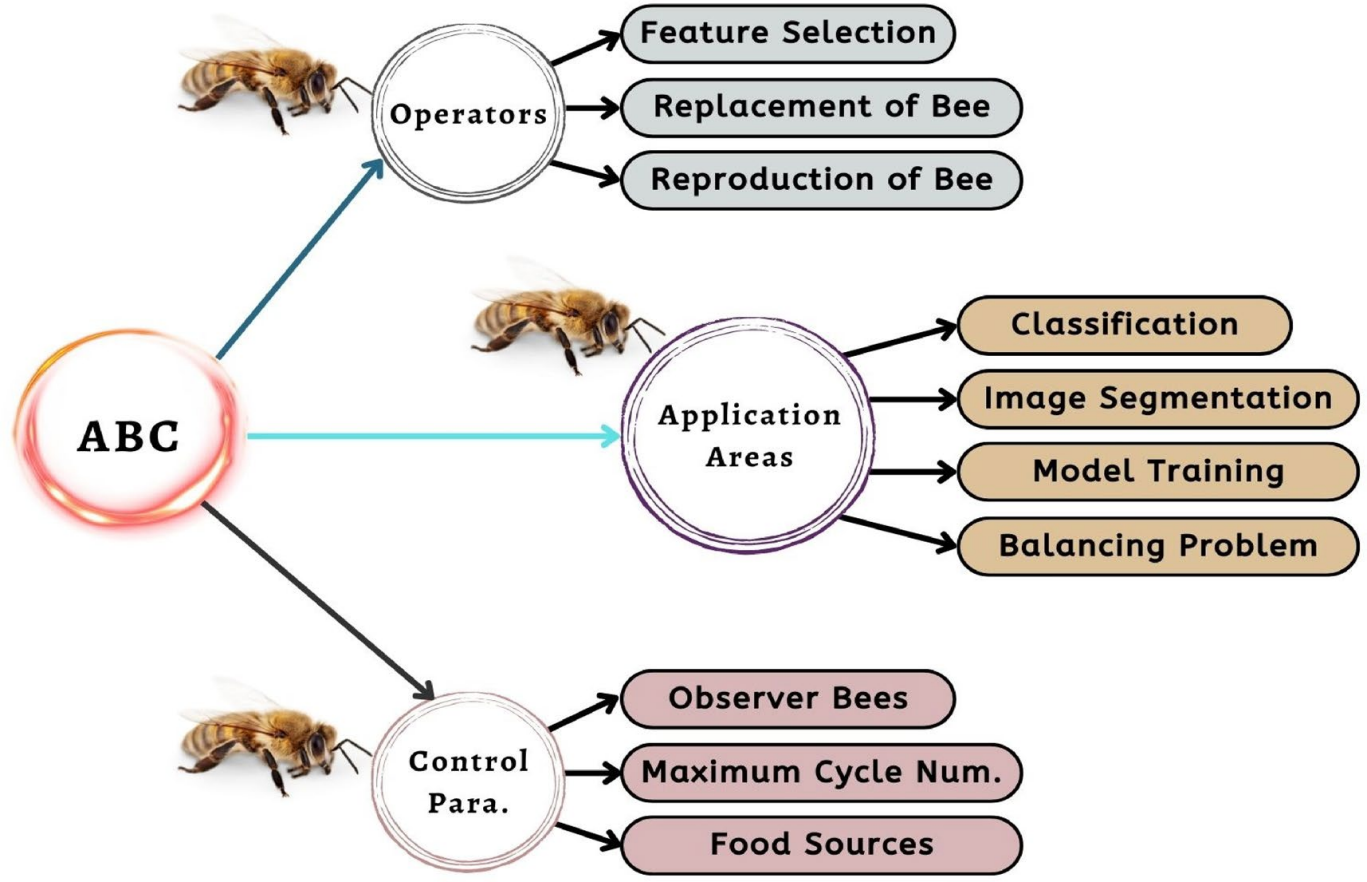
Overview of artificial bee colony (ABC) algorithm.

**Fig. 9:**
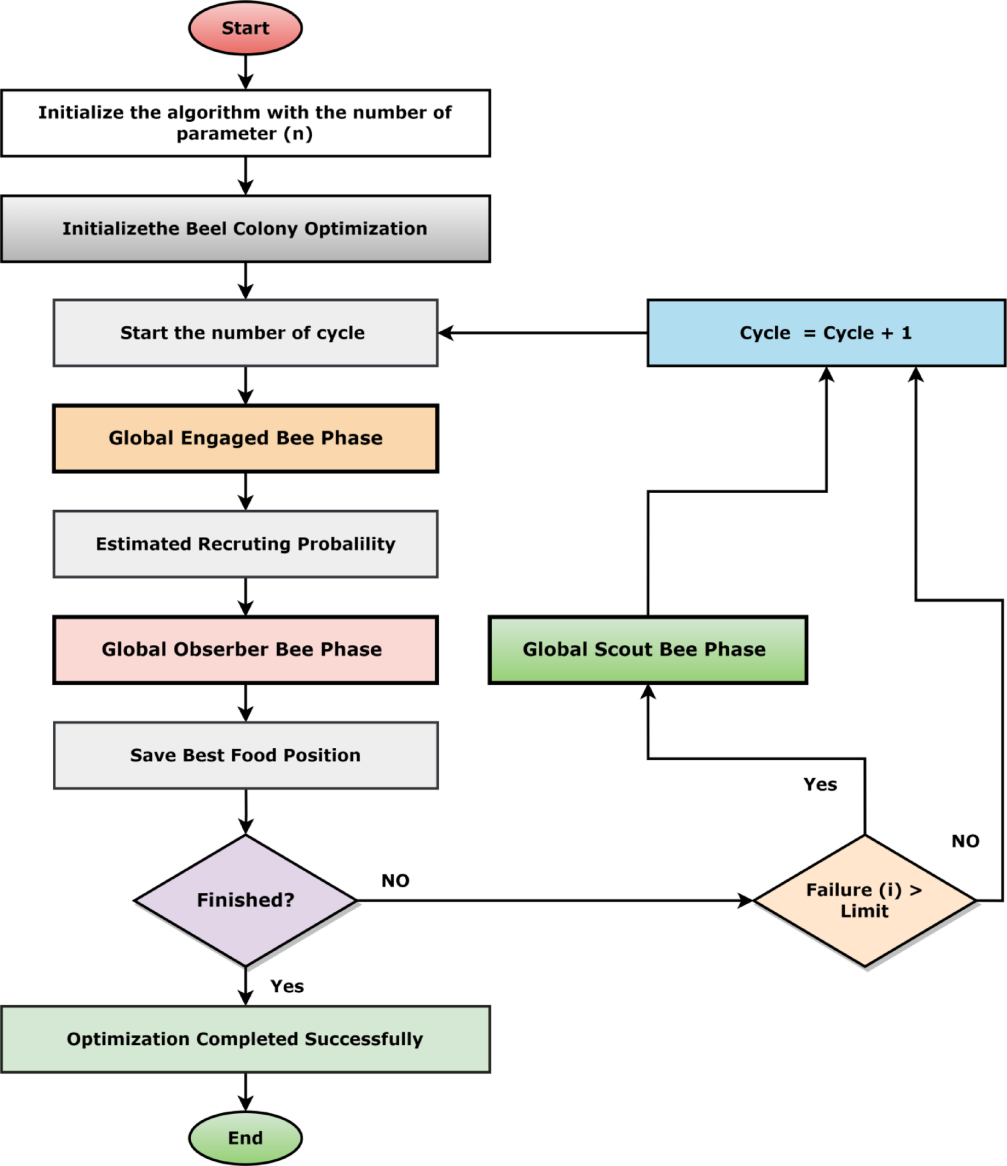
Overview of artificial bee colony (ABC) algorithm.

This research has applied the Artificial Bee Colony (ABC) to optimize the detection of the Locust. ABC algorithm is mainly based on swarm intelligence. This algorithm has executed the tasks better than other stochastic algorithms^26^. The ABC algorithm was selected for feature selection due to its strong balance between exploration and exploitation, simplicity with fewer control parameters, and proven effectiveness in high-dimensional feature spaces. Its lightweight nature also made it highly suitable for integration with our IoT and DL/ML-based framework. The ABC algorithm is determined by a collection of honeybees’ sophisticated foraging activity. ABC has three different types of agents: worker bees, observer bees, and scout bees. The molecule’s subdivided adjacency matrix represents these bees. The algorithm passes through three stages: engaged, observer, and scouting. During the engaged phase, a collection of viable options is generated by modifying one group at a time. During the observer phase, observer bees analyze these solutions and rank them from best to worst based on their significant features. Eventually, if a solution is not improved after a given number of iterations, it is abandoned and replaced with a new solution during the scouting phase. The method cycles through these steps until a near-optimal solution is found. The short overview of the ABC algorithm is shown in Fig. 8 and 9 shows the working mechanism flowchart of ABC in this proposed solution.

### The working principles of IoT-based Agribot

**Fig. 10:**
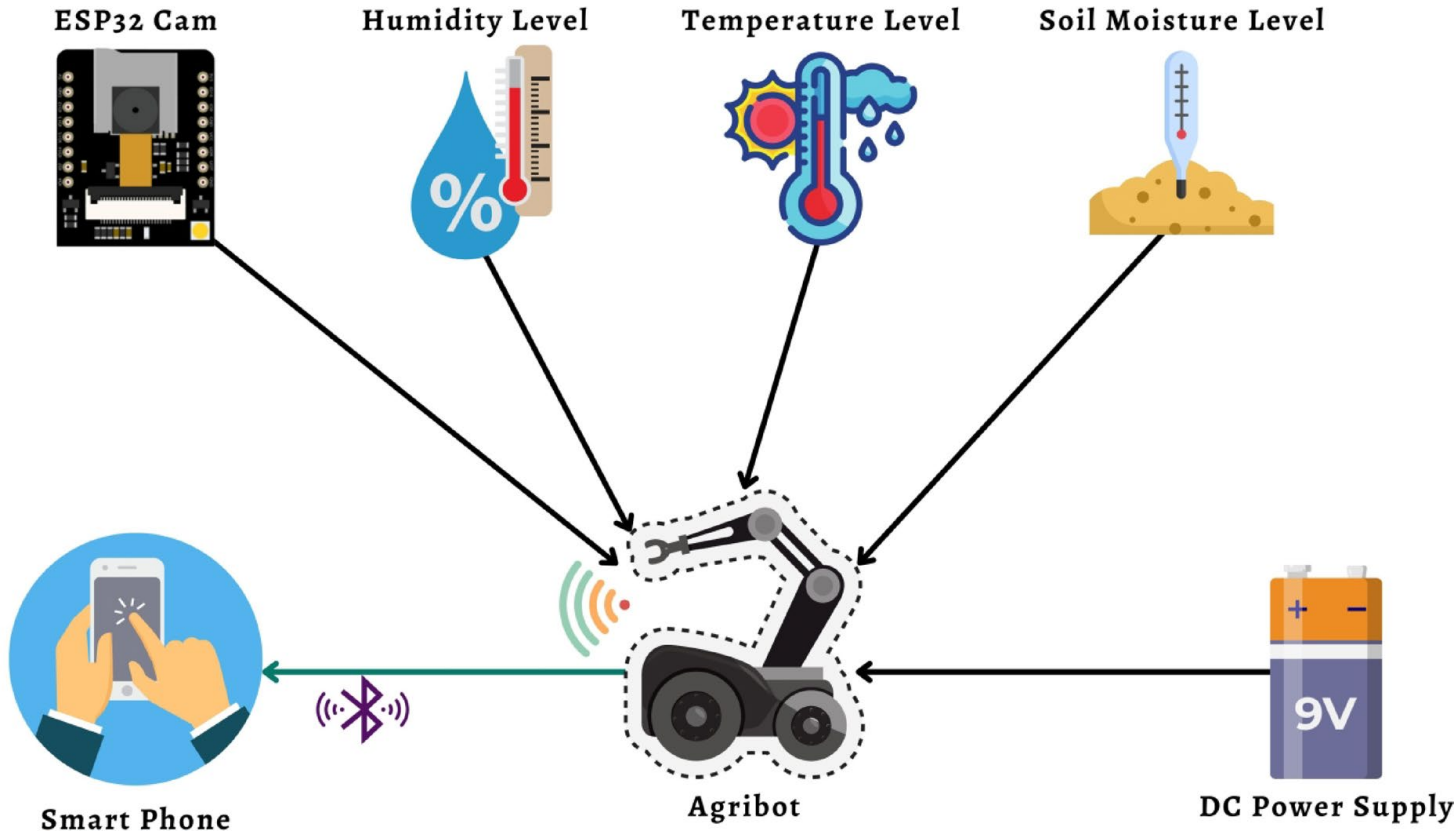
Block diagram of proposed Agribot with multiple sensors and microcontroller.

This subsection presents the working mechanism of Internet of Things (IoT) based Agribot. Figure 10 illustrates the proposed overall diagram of the Agribot. The proposed scheme is implemented with temperature sensors, humidity sensors, soil moisture sensors, and a microcontroller called ESP32 CAM. The Agribot moves through the power supply from 9 V DC power sources. The ESP32 CAM module captures live streaming and sends the data to Agribot, and Agribot transmits the respective streaming to an Android application via a Wi-Fi connection. A Bluetooth connection is also mounted with the Agribot in case of failure of the Wi-Fi connection. These concurrent connections are responsible for the Agribot’s correct movement and for avoiding oncoming obstacles.

#### Working principles of Agribot

**Fig. 11:**
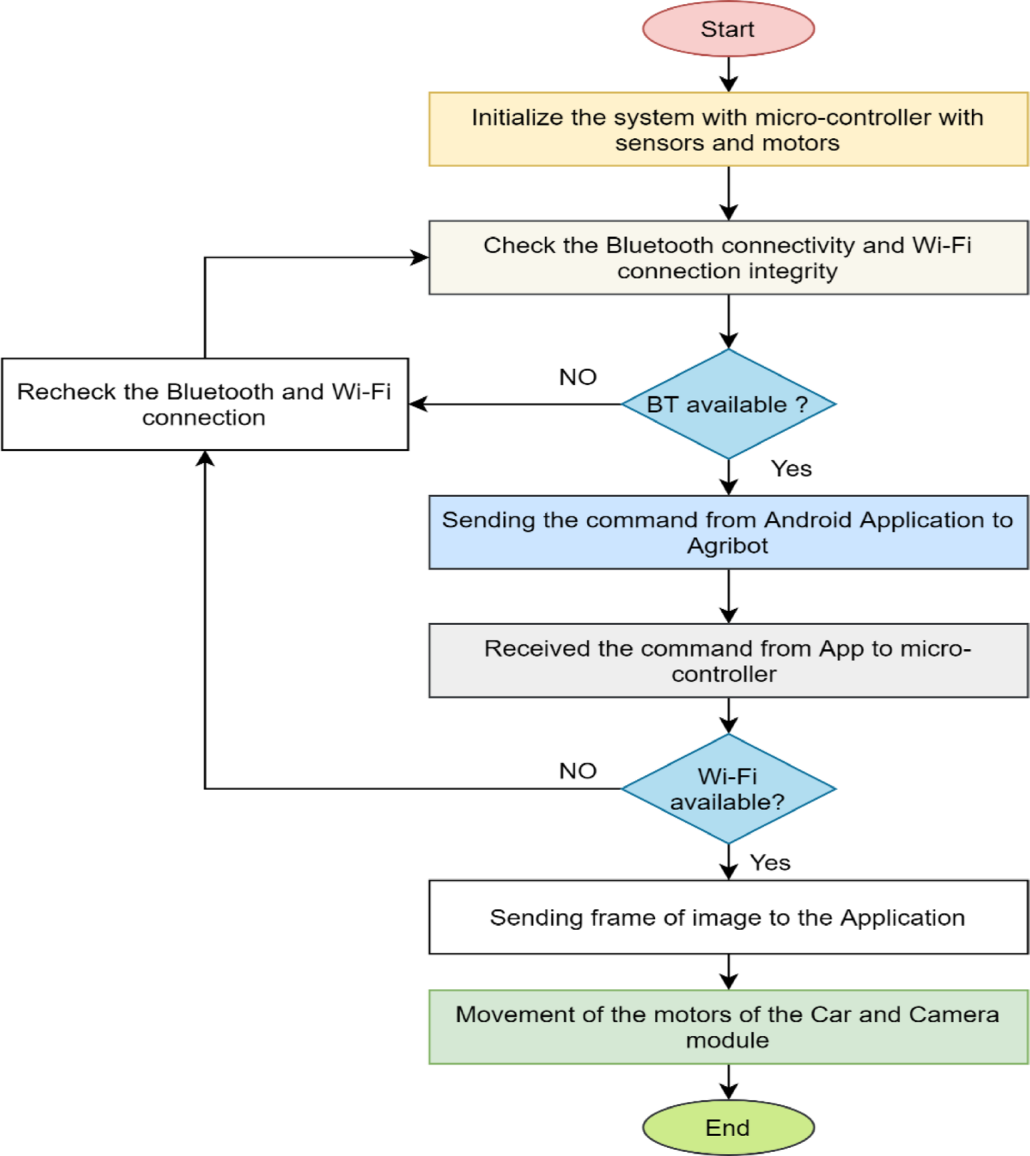
Working principles of Agribot for field automation and movement.

Moreover, Fig. 11 shows the working principle of the proposed Agribot. In this Figure, the system initializes with the microcontroller and multiple sensors. After that, the system checks whether the Bluetooth and Wireless connection is available. If the Bluetooth connection is available, the system can send a command from the android application (app), and the microcontroller receives the command from the app. After that, the scheme checks the wireless connection. The system will send the image frame to ensure real-time video streaming if the wireless connection is also available. An adverse reaction of the Bluetooth and wireless connection will result in a void.

Besides, an algorithm is designed for the movement of Agricultural robots. Algorithm 03 shows the corresponding steps for the right move of the Agribot. In the algorithm, the system takes the upcoming data as input from the Android application. After that, the system defines the analog and digital PINs required for interfacing the sensors with the microcontroller. The scheme defines each particular variable and the threshold value of the speed. A condition ensures the movement of the Agribot in the direction of left, right, forward, and backward positions.

**Algorithm 03:**
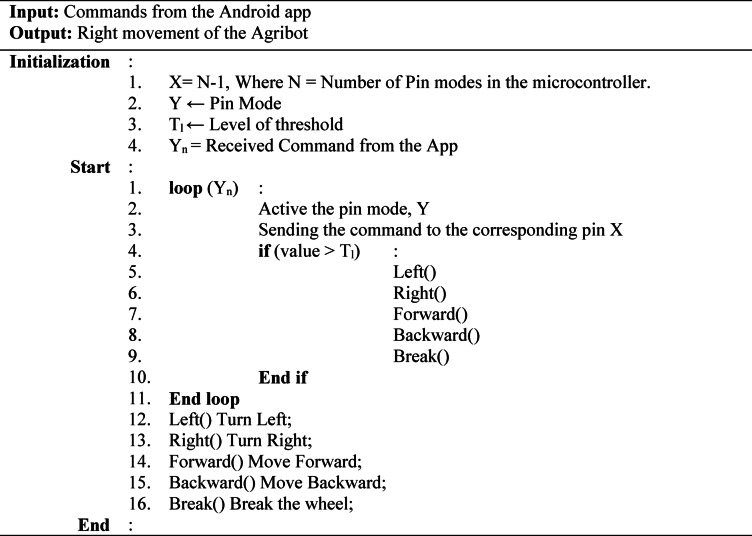
Working mechanism of Agribot for the right movement from the app.

#### Working principles of raspberry Pi and camera module

**Fig. 12:**
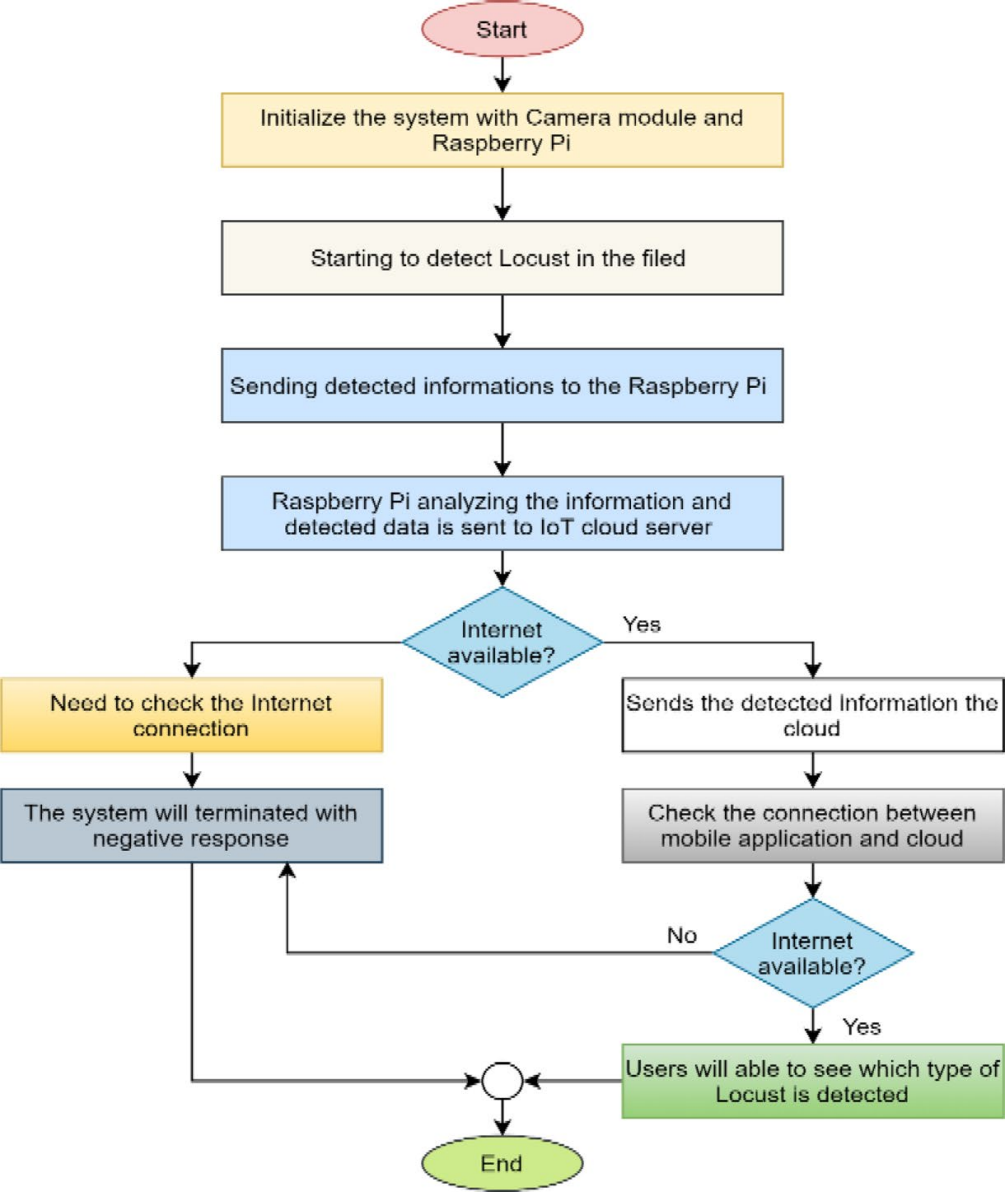
Working principles of Raspberry Pi and camera module to detect locusts in the field.

The Raspberry Pi and camera module detect locusts in the field. Figure 12 shows the working flow chart of the proposed raspberry pi and camera module scheme. The system is initialized in this Figure with the raspberry pi and camera module. After that, the camera starts detecting Locust in the field. If any Locust is found, the system immediately sends the detected information to Raspberry Pi. Then Raspberry Pi analyzes the upcoming information and transmits the image to the IoT cloud server as detected information if the reliable Internet is available. If an internet connection is further available, mobile and IoT cloud server communication can be established. Thus, users can be able to observe the current status of the field in real time. A negative response to the internet connection will result in checking the internet connection, and the system will be terminated with an adverse reaction to the users.

#### Working principles of IoT cloud server

**Fig. 13:**
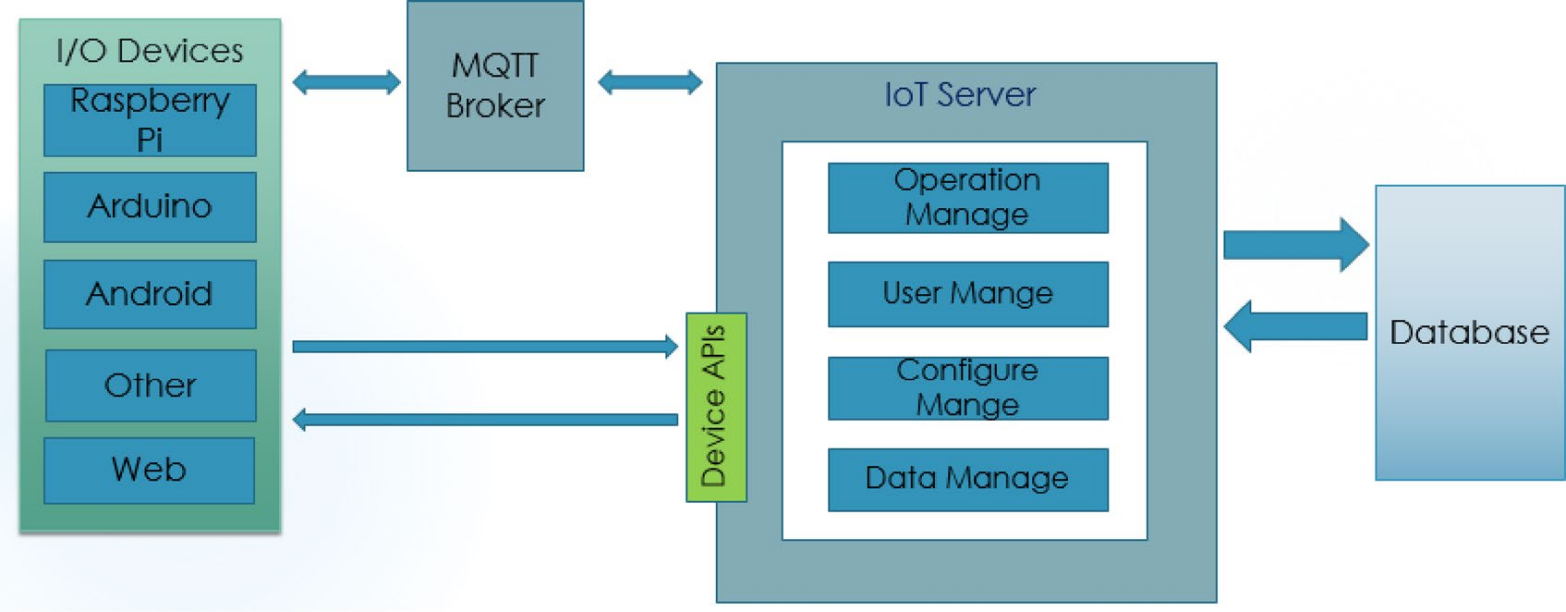
A block diagram of the IoT cloud server of this proposed solution.

This research has designed the IoT cloud server to make the system more reliable. The IoT cloud server is responsible for monitoring the field in real time. Figure 13 shows the corresponding block diagram of the proposed IoT cloud server. This figure can easily track how IoT cloud servers are responsible for sending the data from the sender and server end to the cloud. Message Query Telemetry Transfer (MQTT) broker is placed on maintaining the communication between the IoT cloud server and the sender end^27^. Several operations have been performed in the cloud with the database, like operations management, control of the users, configuration, and data management. The operational process of the IoT cloud server can be categorized into five interconnected parts: registration process, login process, token creation, insect detection, and receiving and showing the data from Raspberry PI.

**Fig. 14:**
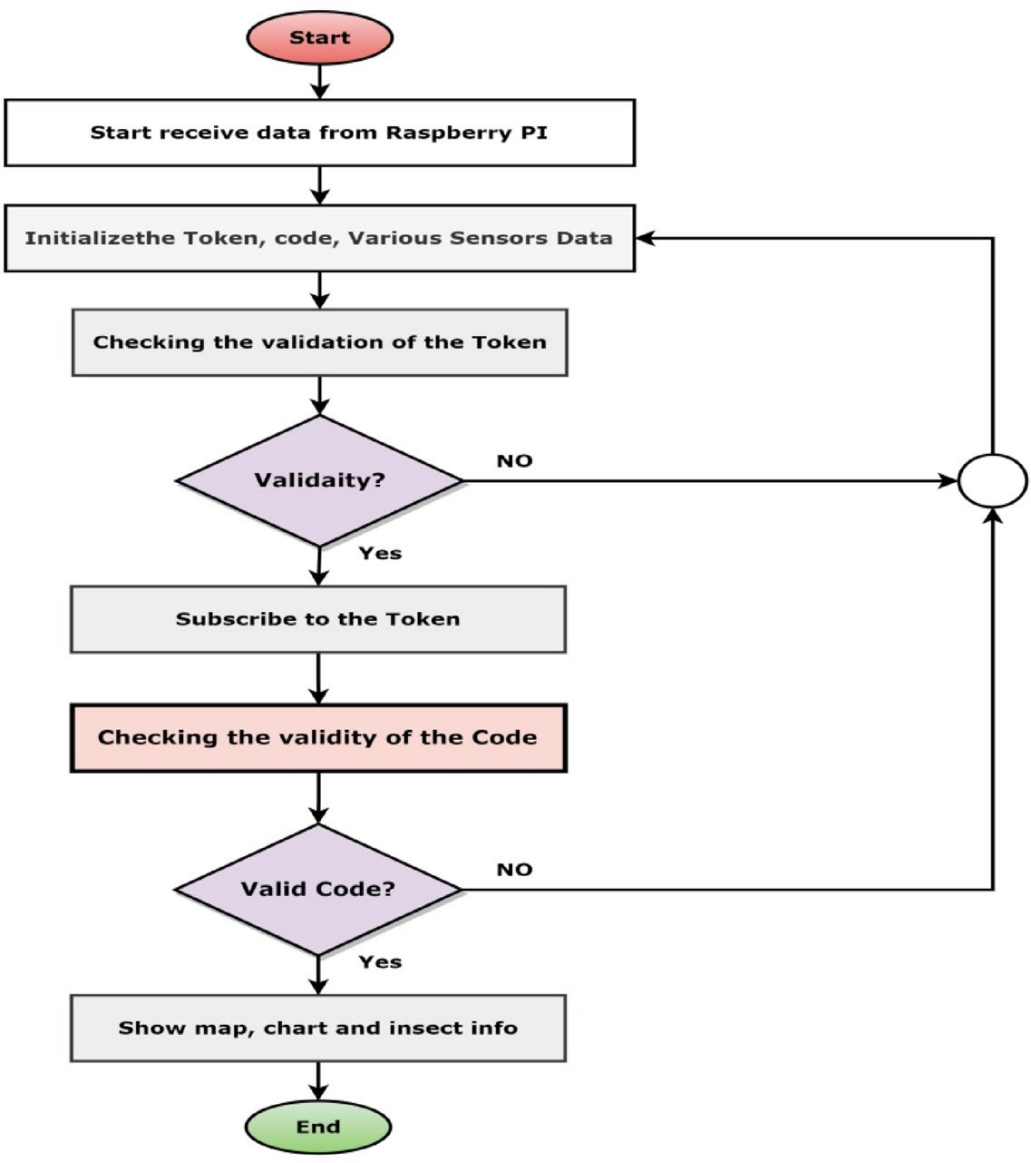
A flow chart of the working principle of receiving and showing data from PI.

To send data to the IoT server from a Raspberry Pi, we have to log in to the IoT server and create a project when we send data to the IoT server. This project data must be sent to the cloud server. Send data from a Raspberry Pi IoT server will be subscribed to the valid token and receive data. This token can also identify the project and the right user and store the data in the database. The IoT server receives humidity, temperature, charge-info, moisture, location, and detected insects code from Raspberry Pi. Those data help us to show gauges, charts, Google Maps, and insect info. Figure 14 shows the corresponding flow chart of the Login process.

## Results and discussion

This section provides experimental data analysis of different modules of Agribot. Firstly, the data analysis with Machine Learning (ML) based models has been enumerated. Secondly, experimental data analysis with IoT-based Agribot is depicted. Thirdly, the System Usability Scale (SUS) score of this proposed method has been presented. Fourthly, this section presents a comparison among existing solutions based on their significant features. Finally, the developed prototype’s snapshots have been illustrated with proper annotations.

### Data analysis with ML-based pipeline

This subsection interprets the ML-based results associated with our developed solution. To examine the findings of this research, a set of matrices are required to measure, such as Accuracy (A), Precision (P), Recall (R), and F1-Score (F1)^21^. The mathematical interpretation of these models is presented in Table 3.

**Table 3:** Description of performance evaluation matrices.

Metrics	Description
Accuracy (A)	Shows the overall exact prediction percentage. $\:\text{A}=\frac{TP+TN}{FP+TP+FN+TN}\times\:100$
Precision (P)	Defines as a way to compute a model’s quality. $\:\text{P}=\frac{TP}{TP+FP}\times\:100$
Recall (R)	Describes as a measurement of model quantity. $\:\text{R}=\frac{TP}{TP+FN}\times\:100$
F1-score (F1)	Shows how reliable and accurate a model is. $\:\text{F}1=2\times\:\frac{R\times\:P}{R+P}\times\:100$

#### Data analysis with pre-trained CNN without artificial bee colony (ABC)

This subsection presents data analysis with Pre-trained CNN models only. This research runs all the associated coding on Google Colab with 53GB RAM and dedicated Graphical Processing Unit (GPU). The subscription of this setup was ‘pro subscription’. The research first extracted the significant features from a particular image. Then the extracted were fed to ML based models to measure performance. In our research, we have spitted the dataset into 80% training data and 20% testing data.

**Fig. 15:**
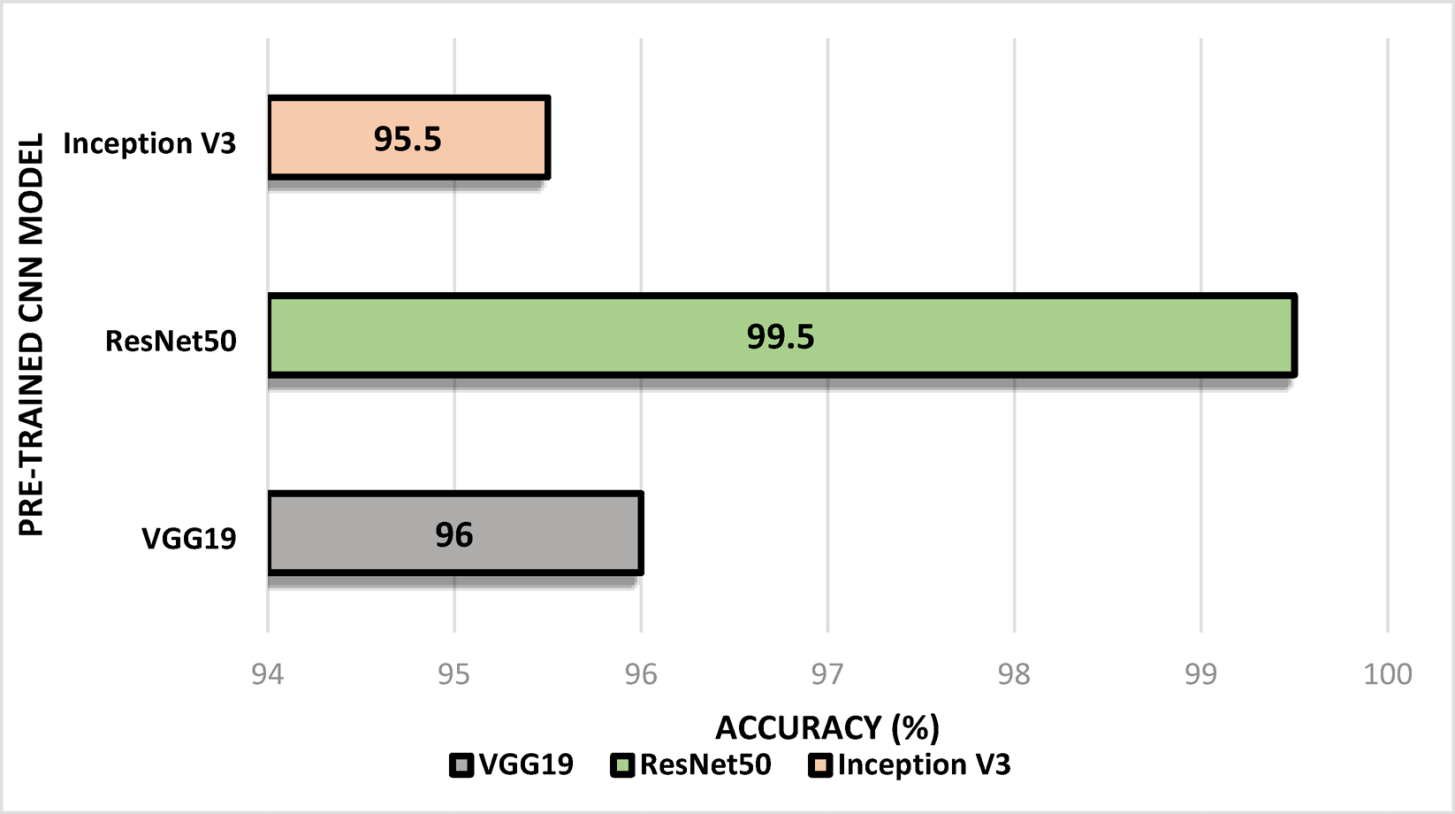
Comparison of accuracy of different CNN models.

We first experimented with the Support Vector Machine (SVM) classifier and pre-trained Convolutional Neural Networks (CNN) architecture without explicitly using the Artificial Bee Colony (ABC) algorithm and Support Vector feature selectors. We have found the highest accuracy of 99.50% in locust detection while dealing with the ResNet50 architecture and SVM classifier. Table 4 shows the corresponding experimental data analysis with the pre-trained models. Also, we have enumerated a comparison among the findings from different models. Figure 15 shows the corresponding bar chart based on the highest accuracy achieved. We have also performed the 5-fold cross-validation for each model to check whether the model discarded the overfitting issues. Table 5 shows the respective summary of findings.

**Table 4:** The overall performance of different Pre-trained CNN models.

CNN model	A	*P*	*R*	F1
VGG19	96.00	95.99	96.03	96.00
ResNet50	***99.50***	***99.52***	***99.47***	***99.50***
Inception V3	95.50	95.50	95.54	95.50

**Table 5:** The fold-wise performance measurement of the different Pre-trained CNN models.

CNN model	Fold 1	Fold 2	Fold 3	Fold 4	Fold 5
VGG19	95.00	95.52	97.00	96.49	95.50
ResNet50	97.98	98.98	99.48	98.48	98.48
Inception V3	95.50	95.52	97.00	96.49	96.51

In addition, we have implemented seven traditional machine learning classifiers, namely Support Vector Machine (SVM), Random Forest (RF), Decision Tree (DT), Naive Bayes (NB), Extreme Gradient Boosting (XGB), K-Nearest Neighbor (KNN), and Logistic Regression (LR). We have analyzed the results in a step-by-step manner, using the performance matrices as a basis for our interpretation. In our finding, we achieved the highest accuracy, 98%, with the LR classifier in locust detection. Also, we have found the highest accuracy of 99.50% with the SVM classifier. Father, we performed the 5-fold cross-validation on each classifier and interpreted their results accordingly. Table 6 summarizes seven classifiers with the VGG19 model, and Table 7 the respective cross-validation results adopted from the VGG19. Nevertheless, Tables 8 and 9 summarize the results of the ResNet50 model and cross-validation.

**Table 6:** The overall performance of different classifiers with VGG19.

Classifiers	A	*P*	*R*	F1
SVC	96.00	95.99	96.03	96.00
**RF**	96.50	96.51	96.48	96.50
DT	92.50	92.49	92.51	92.50
NB	91.50	91.49	91.51	91.49
XGB	97.00	97.00	97.00	97.00
KNC	92.50	93.14	92.33	92.45
LR	98.00	97.99	98.03	98.00

**Table 7:** The fold-wise performance of different classifiers with VGG19.

Classifiers	Fold 1	Fold 2	Fold 3	Fold 4	Fold 5
SVC	95.00	95.52	97.00	96.49	95.50
RF	96.00	96.01	96.00	95.50	95.51
DT	90.00	85.02	85.00	89.49	84.46
NB	88.00	84.49	89.49	88.99	90.00
XGB	94.00	95.00	93.49	94.50	91.99
KNC	85.50	85.51	86.50	86.50	96.51
LR	94.00	94.50	96.00	94.00	94.01

**Table 8:** The overall performance of different classifiers with ResNet50.

Classifiers	A	*P*	*R*	F1
SVC	99.50	99.52	99.47	99.50
RF	98.99	99.06	98.95	98.99
DT	96.48	96.58	96.41	96.47
NB	97.99	97.99	97.99	97.99
XGB	97.49	97.51	97.46	97.48
KNC	98.49	98.47	98.51	98.49
LR	98.99	98.99	98.99	98.99

**Table 9:** The fold-wise performance of different classifiers with ResNet50.

Classifiers	Fold 1	Fold 2	Fold 3	Fold 4	Fold 5
SVC	97.98	98.98	99.48	98.48	98.48
RF	95.48	94.97	95.48	96.98	96.49
DT	91.45	91.95	91.96	93.47	92.98
NB	91.45	91.96	91.98	92.48	92.97
XGB	93.97	93.47	95.47	95.98	95.49
KNC	96.48	97.48	97.48	97.48	96.98
LR	97.98	98.48	99.48	98.48	98.98

**Fig. 16:**
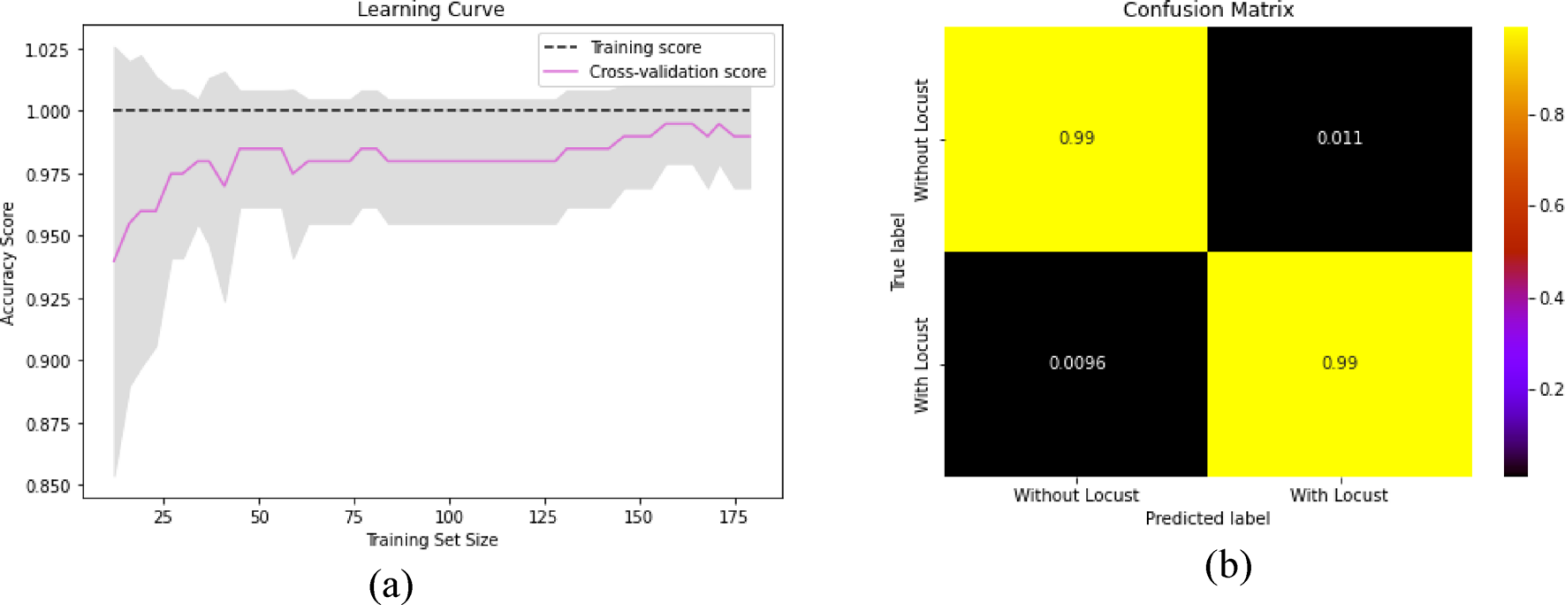
The performance measurement of ResNet50 + SVC (**a**) Learning curve, (**b**) Confusion Matrix.

We have illustrated the learning curve and confusion matrix on the sharp contrast for the highest accuracy from Restnet50 and SVM classifiers. Figure 16(a) shows the learning curve, and Fig. 16(b) shows the locust detection confusion matrix.

#### Data analysis with pre-trained CNN without ABC and SVC feature selectors

We have performed ABC algorithm with SVC feature selectors to work with more optimized features and test the model performance. With the ABC and SVC, we have found a significant improvement in locust detection accuracy as well in Precision, Recall, and F1-score. Table 10 shows the corresponding summary of the findings. From the table, we can make clearly obverse that the research has found the highest accuracy of 99% while dealing with VGG19 + SVM with the ABC algorithm & SVC feature selector, where the accuracy with VGG19 was 19%. We have found nearly the same result from the ResNet50 model. We have also enumerated the number of best-selected features through the SVC feature selector and ABC algorithm. Further, we performed the 5-fold cross-validation and interpreted the experimental results in Table 11.

**Table 10:** The overall performance of different Pre-trained CNN models with ABC + SVC feature Selector.

CNN model	No. best selected features	Subset accuracy	A	*P*	*R*	F1
VGG19	2077	99.00	99.00	99.01	99.01	99.00
ResNet50	1014	98.99	98.99	99.00	99.01	98.99
Inception V3	1014	98.00	98.00	97.75	97.75	97.50

**Table 11:** The fold-wise performance measurement of the different CNN models with ABC + SVC feature Selector.

CNN model	Fold 1	Fold 2	Fold 3	Fold 4	Fold 5
VGG19	97.50	97.00	98.00	97.00	97.01
ResNet50	97.50	98.00	98.50	98.50	98.51
Inception V3	97.50	97.00	97.75	97.75	97.50

Moreover, we have also performed the seven classifiers on VGG19 along with ABC and SVC feature selectors. In this experiment, we found the highest accuracy of 99.51% in locust detection with the LR classifier. In these experiments, we have discovered that VGG19 features work more efficiently with the LR classifier and ABC algorithm. The summary of the results is depicted in Tables 12 and 13. In addition, we have performed the same experiment with ResNet50 architecture. But with the ABC algorithm and SVC feature selector, the accuracy was nearly 99% with SVM and LR classifier.

**Table 12:** The overall performance of different classifiers with VGG19 with ABC + SVC feature selector.

Classifiers	A	*P*	*R*	F1
SVC	99.00	99.01	99.01	99.00
RF	99.00	99.01	99.01	99.00
DT	94.50	94.55	94.48	94.50
NB	97.00	97.00	97.00	97.00
XGB	98.00	98.06	98.02	98.00
KNC	96.00	96.26	96.04	96.00
LR	99.51	99.50	99.50	99.50

**Table 13:** The overall performance of different classifiers with ResNet50 with ABC + SVC feature selector.

Classifiers	A	*P*	*R*	F1
SVC	99.00	99.01	99.01	99.00
RF	98.49	98.51	98.51	98.49
DT	93.97	94.21	94.03	93.97
NB	96.98	97.02	96.97	96.98
XGB	98.99	99.00	99.01	98.99
KNC	98.49	98.51	98.51	98.49
LR	98.99	99.00	99.01	98.99

**Fig. 17:**
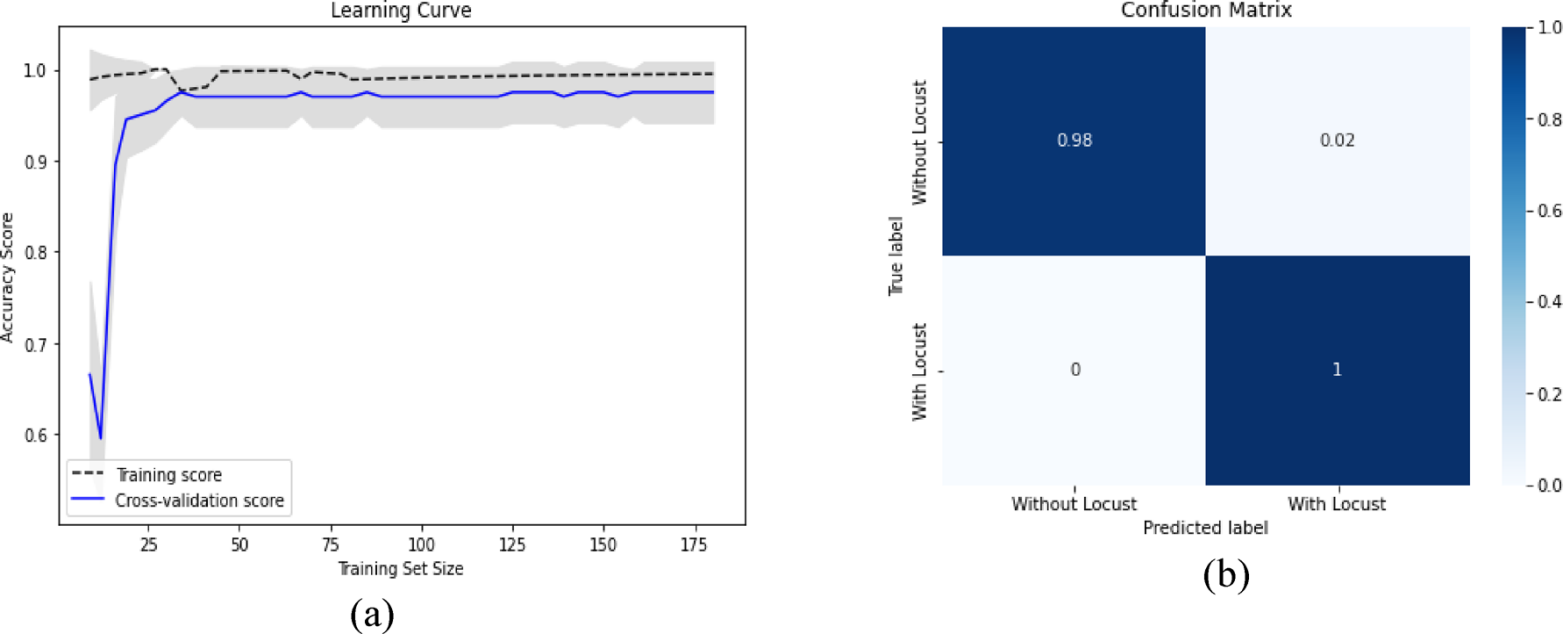
The performance measurement of VGG19 + SVC + ABC with the (**a**) Learning curve, (**b**) Confusion Matrix.

On the other hand, the learning curve and confusion matrix for the highest accuracy VGG19 and LR classifier, along with the ABC algorithm and SVC feature selection, are shown in clear contrast. Figure 17(a) displays the learning curve, and Fig. 17(b) depicts the confusion matrix for locust detection.

#### Comparison among different techniques

**Fig. 18:**
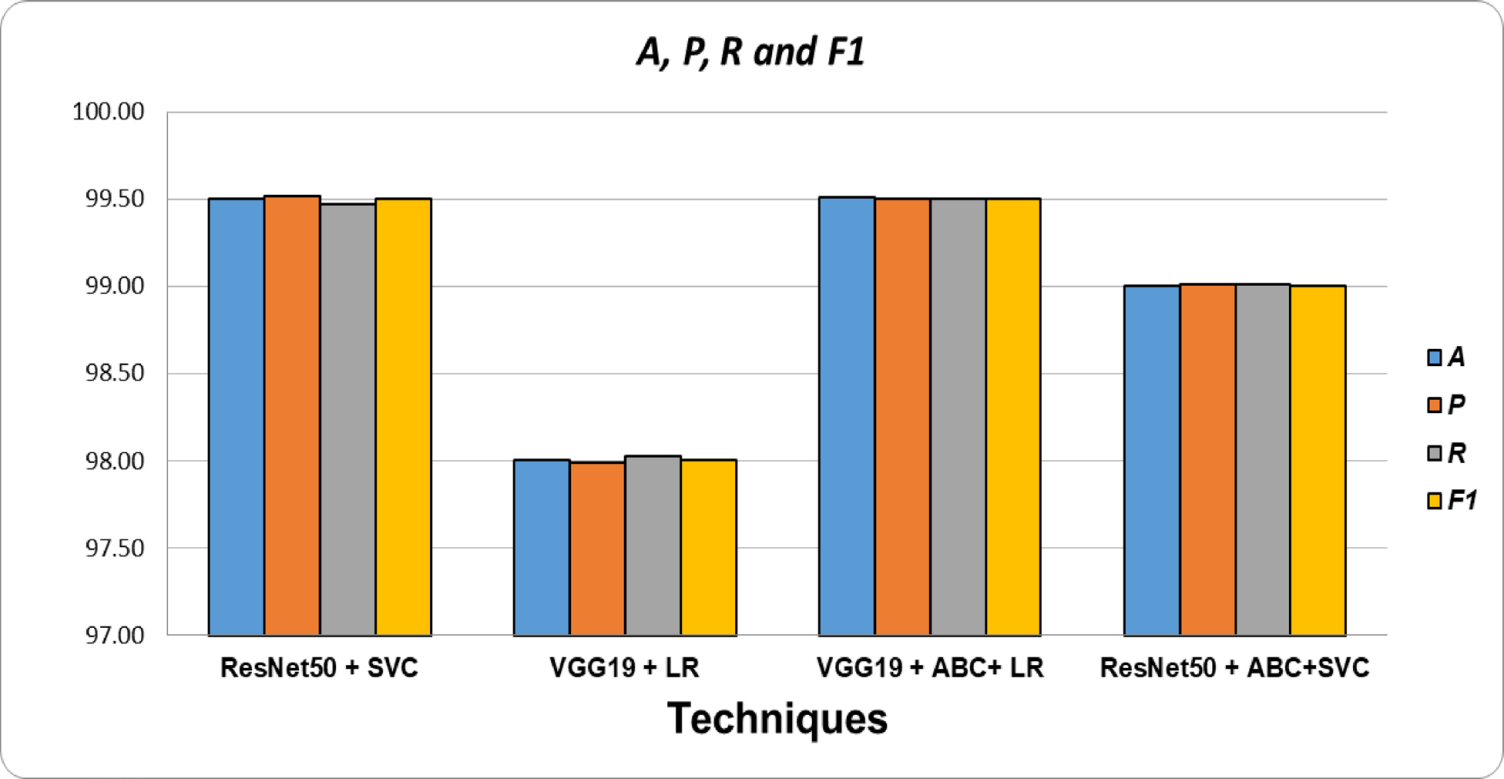
Comparison of accuracy of different techniques.

In this study, we have also performed the comparison among the highest accuracy in locust detection with different types of techniques. Table 14 shows the corresponding comparison among the various techniques or combinations in our result analysis. Besides, Fig. 18 displays the performance comparison bar chart and performance evaluation matrix. This figure clearly illustrates that Resnet50 with SVC and VGG19 with ABC & SVC give the highest result in locust detection compared to the other models.

**Table 14:** The overall performance of different classifiers with ResNet50 with ABC + SVC feature Selector.

Techniques	A	*P*	*R*	F1
ResNet50 + SVC	99.50	99.52	99.47	99.50
VGG19 + LR	98.00	97.99	98.03	98.00
VGG19 + ABC + LR	99.51	99.50	99.50	99.50
ResNet50 + ABC + SVC	99.00	99.01	99.01	99.00

### Experimental data analysis with IoT-based agribot

This research has utilized several agricultural sensors. We have mounted them in the robot and calculated them accordingly. In our experiments, we calculated the data of each particular sensor in the same experiment. Table 15 shows the corresponding data analysis of each sensor. Data calculation is taken place on our university premises.

**Table 15:** Sensor data calculation of agricultural sensors.

Serial no.	Temperature (°C)	Humidity level	Soil moisture level	The decision on data calculation
1	28	98	450	Soil is dry, temperature 28 °C, humidity 98
2	28	96	590	Soil is almost dry, the temperature is constant, and the humidity changed
3	28	96	600	Soil is almost dry, temperature constant, and humidity the same as before
4	28	97	800	Soil is almost wet, the temperature is constant, and the humidity changed
5	28	97	850	Soil is almost wet, temperature constant, and humidity the same as before
6	28	96	522	Soil is almost dry, and the temperature is constant, humidity changed
7	28	96	600	Soil is almost dry, temperature constant, and humidity the same as before
8	28	96	650	Soil is wet, temperature constant, and humidity the same as before
9	28	96	820	Soil is wet, temperature constant, and humidity the same as before

**Fig. 19:**
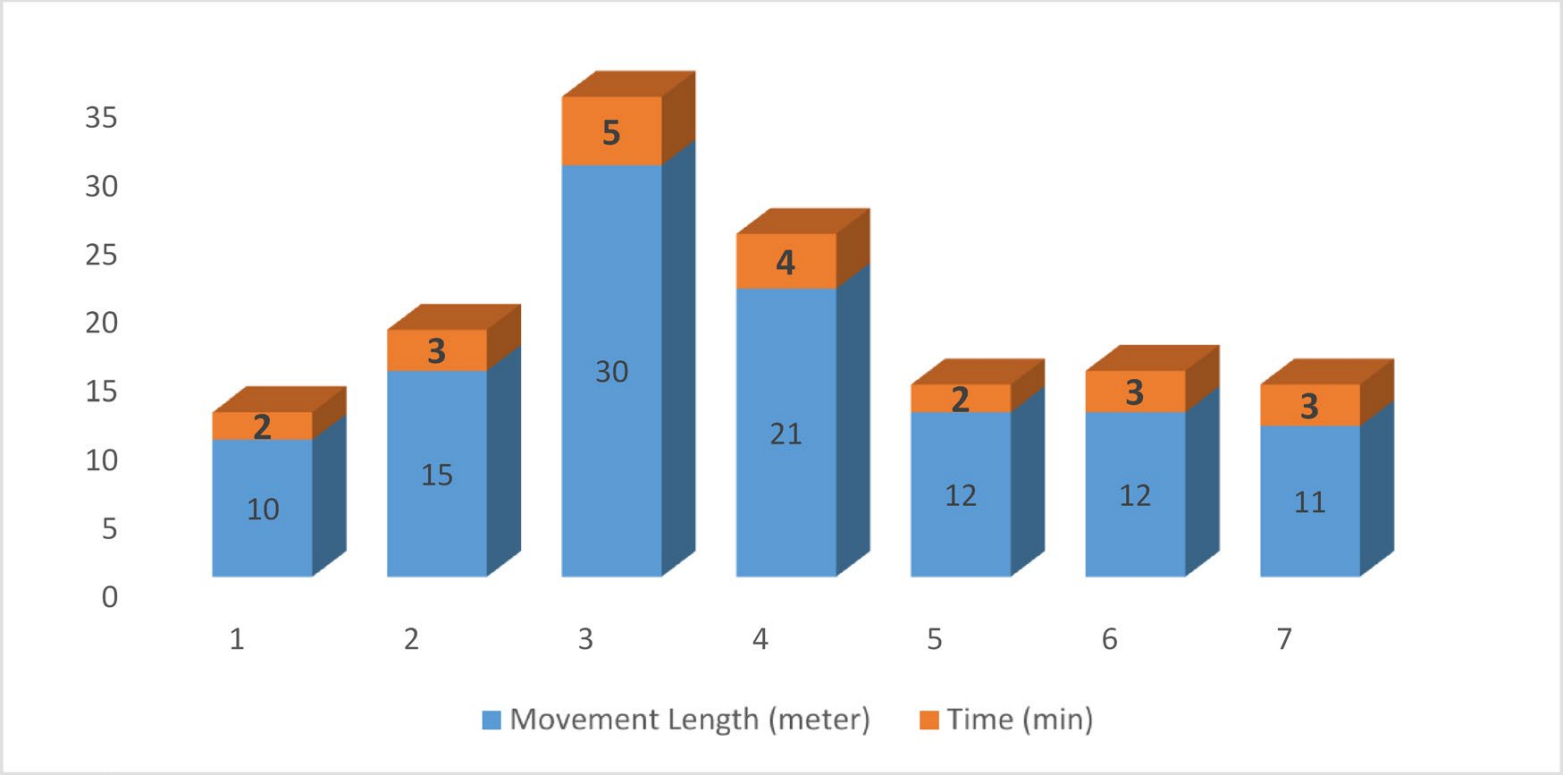
Agribot movement vs. the required time.

From this table, we can easily track out that the temperature in the field remained unchanged due to the environment’s nature. The humidity level changed as the humidity level changed in the air. Again, we examined soil with dry soil and wet soil and found the sensor’s moisture level accordingly. We have driven the robot with our developed mobile application. A full 9-volt battery charge gives backup for up to One and a half hours. We have tracked an average speed up to 0.3048 m/s or 1.09728 km/h. Table 16 presents the experimental data analysis of Agribot. In the table, we can easily track the movement of the Agribot. We have performed our experiment with maximum movement length and direction criteria. We have found the greatest range of motion of Agribot is 30 m, and the maximum time required for the maximum range of movement is 5 min. Our experiment tracked our average movement of 16 m within 2 min. We observe that the necessary time is scattered from the mean value because of the path’s shape and size. Again, live video streaming is disconnected or held at speed when increasing its peak value. Figure 19 shows the corresponding comparative result achieved from the Agribot.

**Table 16:** Experimental data analysis of Agribot.

Experiment no.	Movement length (meter)	Direction criteria (Left-L, Right-*R*, Forward-F, Backward-B)	Time (min)	Send to the cloud (yes/no)	Live video streaming status (connected/disconnected)
1	10	F–R–L–B	2	Yes	Connected
2	15	F–L–R	3	Yes	Connected
3	30	F–L–R	5	Yes	Disconnected
4	21	F–L–R	4	No	Disconnected
5	12	F–L–R–B	2	Yes	Connected
6	12	B–L–R	3	Yes	Connected
7	11	B–R–L	3	Yes	Connected
Average	16 m				

### System usability scale (SUS)

**Fig. 20:**
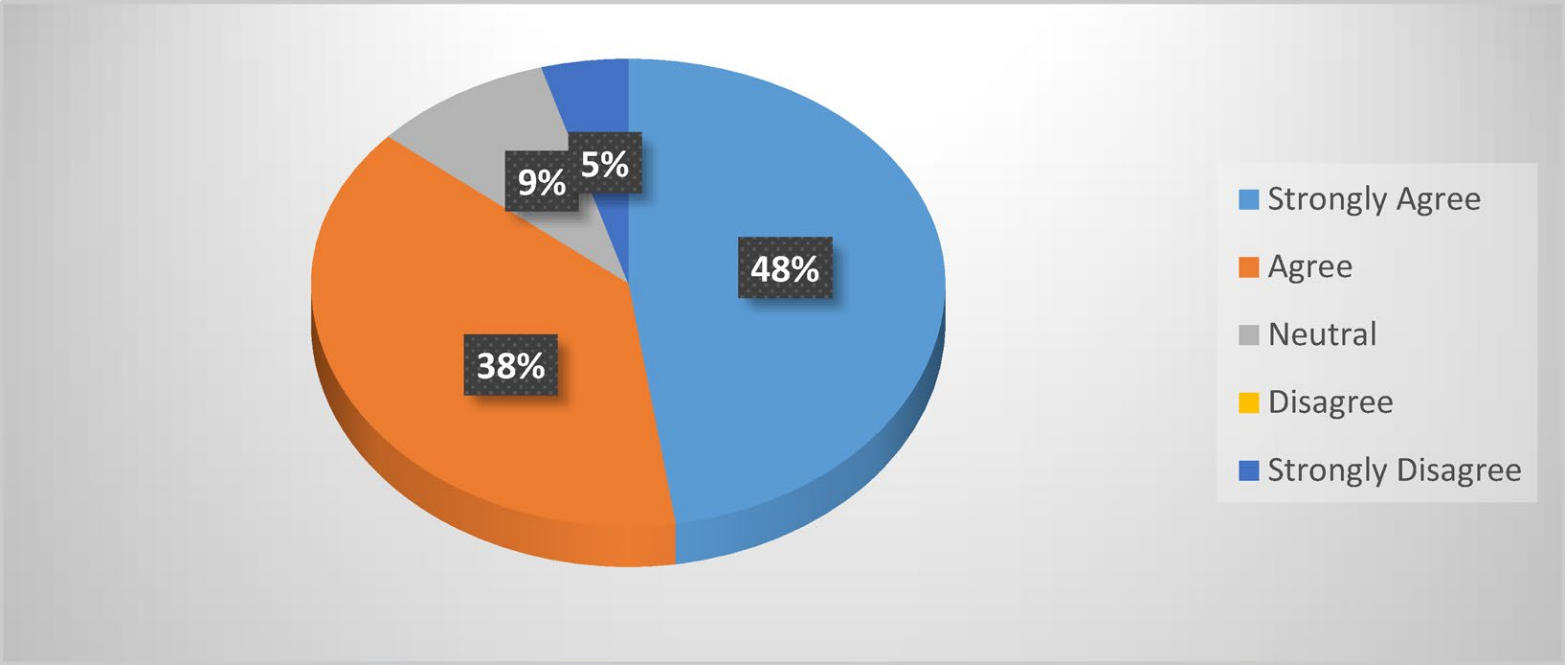
Experimental data of system usability scale (SUS).

In our established model, we have carried out a System Usability Scale (SUS) among ten randomly selected individuals. We have provided them with our developed mobile application and Agribot. They had driven our robot through the progressive mobile application for up to 10 min. After that, we took their opinion on our developed application. We also found the product of appeasement from the consumers. 48% of users strongly agree with the SUS ranking, and 38% of users agree with our work. This outcome shows that 86% of individuals recommend our established model, 9% remain neutral, and 5% strongly refuse our proposed method. The corresponding experimental data is illustrated in Fig. 20.

### Comparison among the existing solution

The research also ensures a comparison among existing papers. We have compared the literature with our developed Agribot on methodology, contributions, and highest accuracy regarding Machine Learning (ML) models and features. Table 17 shows the corresponding comparison table extracted from the literature review.

**Table 17:** Comparison among related systems with our developed scheme.

Referenced papers	Methodology	Deep/machine learning (yes/no)	Highest accuracy	IoT based system (yes/no)	Robot development (Yes/No)	Remarks
9	Machine learning approaches utilized in insect detection	Yes	45%	No	No	Partially matched with our developed scheme
10	IoT-based technology has been established to detect and monitor insects.	No	N/A	Yes	No	Partially matched with our developed scheme
11	Machine learning approaches used to detect and analyze mosquitoes	Yes	99.9%	No	No	Partially matched with our developed scheme
13	A machine learning model has been adopted to classify locusts.	Yes	97.80%	No	No	Partially matched with our developed scheme
14	Developed a robot to water the plant automatically	No	N/A	No	Yes	Partially matched with our developed scheme
15	Agribot has been developed for seed sowing with IoT-based technology.	No	N/A	Yes	Yes	Overwhelmingly matched with our developed scheme
17	Machine learning techniques adopted to detect and predict insects	Yes	98.60%	No	No	Partially matched with our developed scheme
Our developed system	Developed Agribot using IoT and Machine learning to detect and analyze locust	Yes		Yes	Yes	The combination of three fields

### Snapshots of the developed prototype

This research ensures the development process of Agricultural robots utilizing Android-based hardware development and IoT cloud development. We first developed the Agricultural robot using the Arduino Mega board, motors & motor driver, and multiple agricultural sensors in our development process. After that, we designed the mobile application of the proposed methodology through the android studio platform. Finally, we look forward to developing the proposed IoT server in response to the proposed architecture. In the following sections, we present the snapshot and screenshot of the developed solution and its caption (see Supplementary Appendix A).

## Conclusion and future work

In several African and Middle Eastern nations, locusts have significantly harmed agricultural production, inflicting massive economic loss. One possible solution for this problem is an agricultural robot (Agribot). In contrast, the Internet of Things (IoT) plays an important role in agriculture due to its promising features, such as real-time data monitoring and deep learning processes, which lead to an agile solution in object detection, such as insect categorization and recognition. This work demonstrates an intelligent strategy for designing an Agribot using IoT in combination with Machine Learning (ML) and Deep Learning (DL) based architecture for locust identification in the agricultural area. The IoT component enables correct automation by employing numerous agricultural sensors, a focused Android application, and an IoT cloud server. The ML and DL technique, on the other hand, integrates certain pre-trained Convolutional Neural Network (CNN) models with traditional ML classifiers, as well as a nature-inspired algorithm such as Artificial Bee Colony (ABC) and SVC feature selection. Experimental data collection has been enumerated and evaluated to assess the efficacy of the suggested orientation. This study achieved the maximum locust detection accuracy of 99.51% using a VGG19 pre-trained CNN model with Logistic Regression (LR) and SVC feature selector. Besides, with live video streaming, The Agribot has moved more effectively and at a reasonable speed in the agricultural field. According to the research, the Agribot’s top speed is up to 0.3048 m/s. Furthermore, the investigation discovered a SUS score of 86% on our proposed method. Although the system performs better in locust identification and automation, the study found certain drawbacks when investigating and executing the idea. Firstly, we faced difficulties moving the bot in the field because the motor had been shut down due to the path’s soil level. Secondly, this has performed the ML and DL based on still images, not real-time video streaming. Thirdly, the proposed model can be implemented inside the raspberry pi without building a model in the cloud.

We will overcome the previously highlighted concerns in the future. According to our plan, we will use Raspberry Pi’s proposed detection mechanism to detect insects with maximum precision. Again, we intend to develop a method to spread seeds and fertilizer using our suggested Agricultural Robot. Also, due to the distance between the sender and receiver ends, live video streaming has been halted. Because LAN technology operates in a small region, video streaming is terminated or held for speed. We will include this in our upcoming modification. We encountered issues moving the Agribot backward because it is only intended in the Left, Right, Forward, and Reverse positions. We anticipate that adding a video camera behind the robot will be useful in resolving this issue. Again, we discovered problems accessing the IoT cloud server’s data via a mobile application. As a result, we want to create a user-friendly mobile application for the next modification. Furthermore, we will create a powerful and efficient database system because the IoT cloud server only focuses on real-time sensor data monitoring. Still, no module has been designed to store and retrieve the incoming data. The suggested technique would also identify illnesses in the leaves of various species of trees. Nonetheless, the proposed technology can monitor locusts in agricultural areas in real-time. Furthermore, we have a plan to add the drone-based system to our proposed model; thus, the locust or insect system will be more effective both in the ground and in the sky monitoring.

## Supplementary Information

Below is the link to the electronic supplementary material.

Supplementary Material 1

## Data Availability

The authors declare that the data supporting the findings of this study are available within the paper, and the Locust Computer Vision Project (https://universe.roboflow.com/khadija/locust-6a5yl).
