# Supplementary material for: An AI-powered smart Agribot for detecting locusts in farmlands using IoT and deep learning
Source: Sci Rep. 2025 Nov 13;15:39848. doi: 10.1038/s41598-025-23497-8 (PMC12615658; doi:10.1038/s41598-025-23497-8)
Supplement: Supplementary file 1 — Supplementary Material 1 [file 41598_2025_23497_MOESM1_ESM.docx]

# **Appendix A**


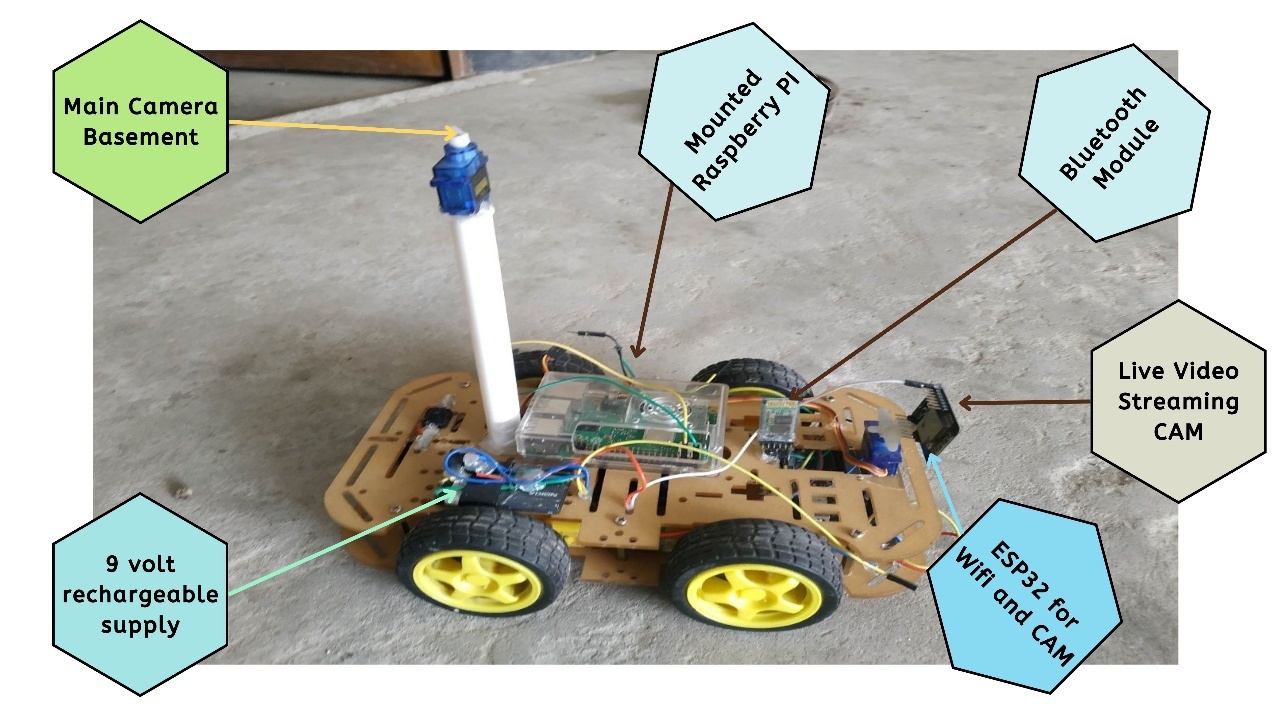


Figure 01: A left-angle view of the developed Agribot with Raspberry PI.


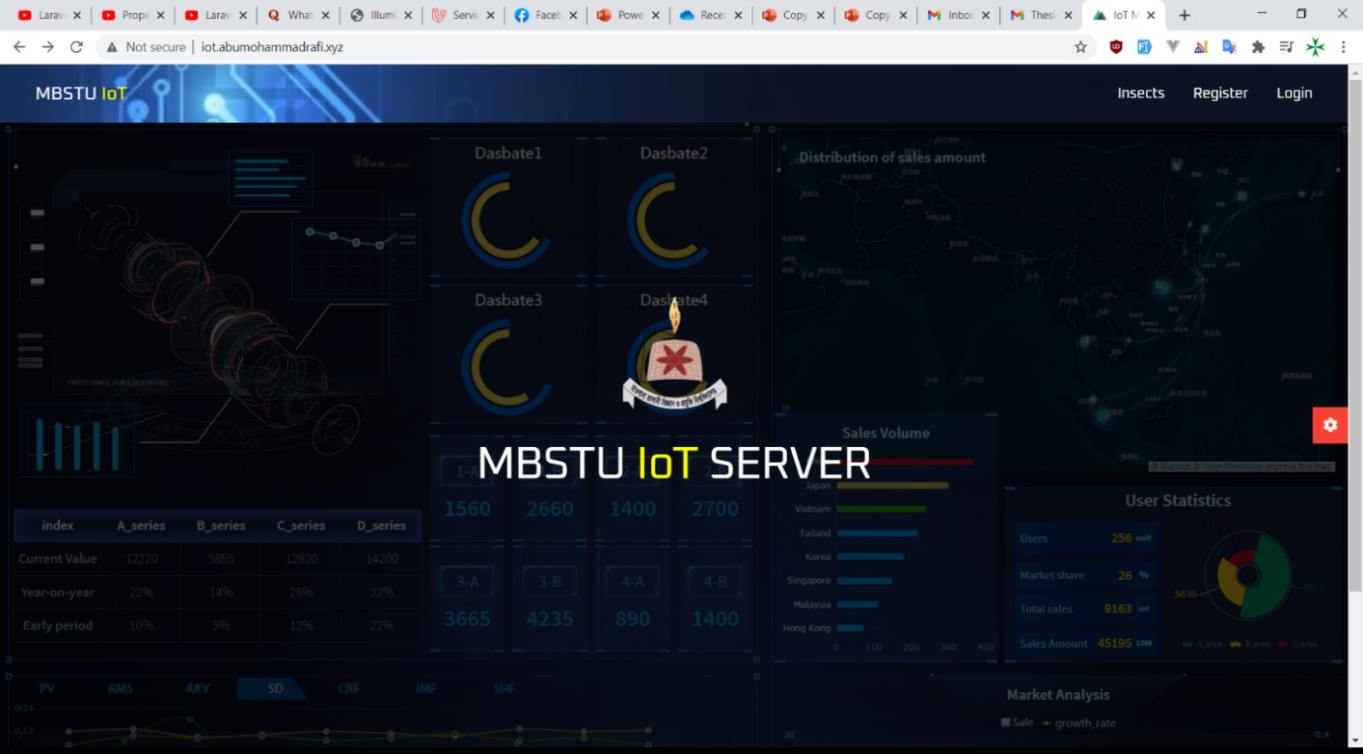


Figure 02: U.I. design of the Home page of the developed IoT server


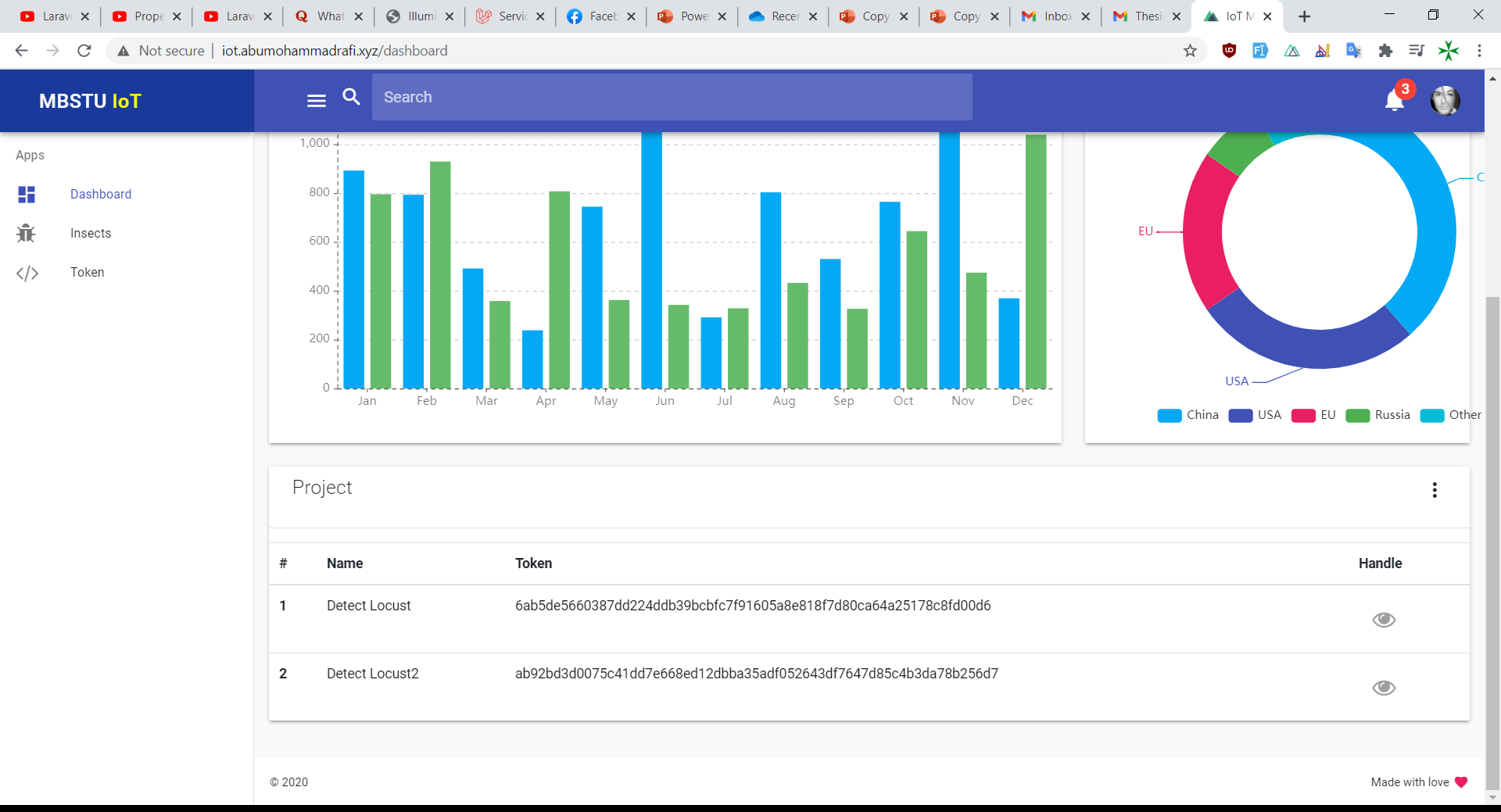


Figure 03: A dashboard of real-time monitoring of Locust


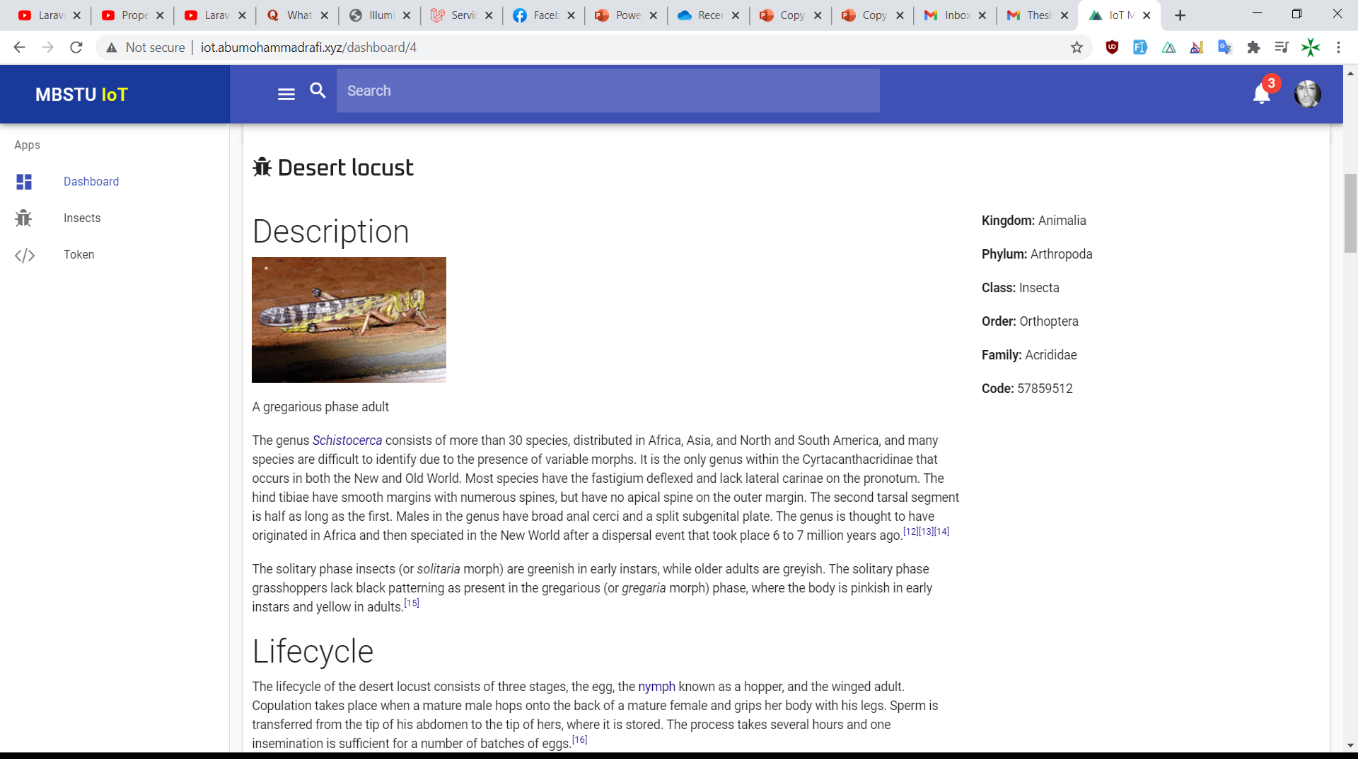


Figure 04: A complete biography of the detected Locust in the field

| 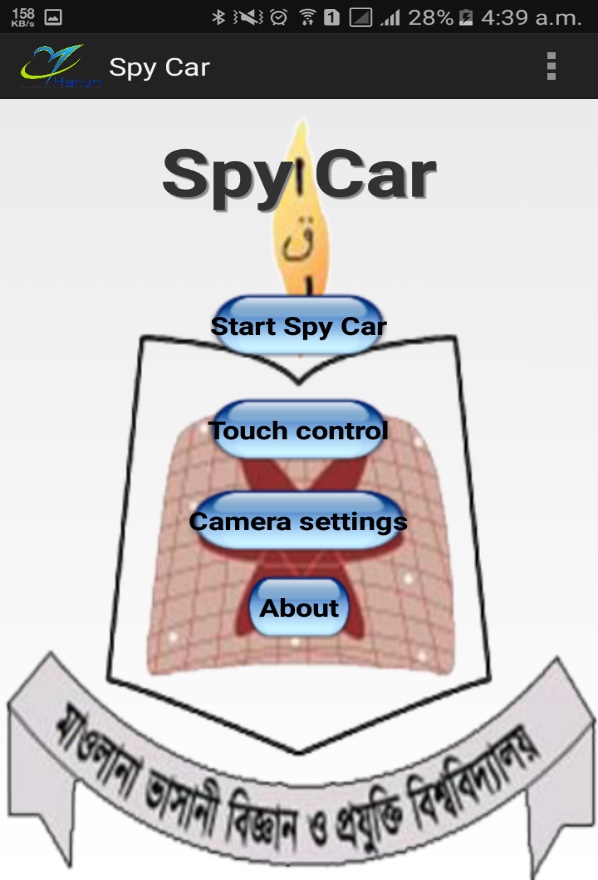 | 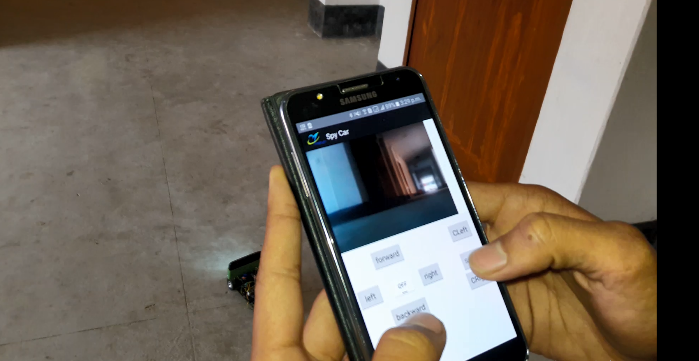  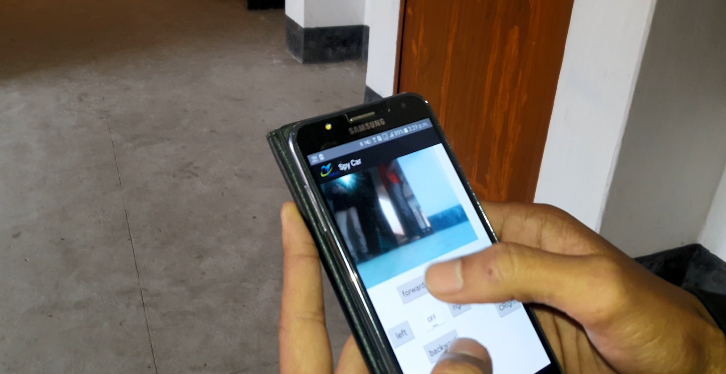 |
| --- | --- |
|  |  |

Figure 05: Screenshots developed for mobile application
